# Phenotypic and Genomic Characterization of Polyethylene-Degrading *Bacillus cereus* PE-1 Enriched from Landfill Microbial Consortium

**DOI:** 10.3390/polym18060695

**Published:** 2026-03-12

**Authors:** Weijun Wang, Shunyu Yao, Zhimin Liu, Xiaolu Liu

**Affiliations:** School of Chemistry and Biological Engineering, University of Science and Technology Beijing, 30 Xueyuan Road, Haidian District, Beijing 100083, China; wangweijun@ustb.edu.cn (W.W.); yaoshunyu3d21@163.com (S.Y.); liuzhimin_1@126.com (Z.L.)

**Keywords:** polyethylene, microplastic biodegradation, *Bacillus cereus*, biofilm formation, oxidative degradation, functional genes

## Abstract

Polyethylene (PE) is one of the most persistent pollutants in the environment. Here, we enriched a microbial consortium (PEH) and isolated a bacterial strain, *Bacillus cereus* PE-1, capable of degrading PE from landfill soil using PE as the sole carbon source. Scanning electron microscopy revealed significant surface erosion, while weight loss reached up to 4.57% after 30 days. TGA showed a 5.88% decrease in onset degradation temperature, and contact angle measurements indicated increased hydrophilicity. Elemental analysis confirmed oxygen incorporation into the polymer matrix. Genome sequencing revealed genes associated with biofilm formation (*epsA*, *epsB*, *pgaC*), oxidation (laccase, copper oxidase), hydrolysis (esterase, lipase, PHB depolymerase), and β-oxidation pathways. While these genomic findings indicate a predicted capacity for assimilation, no transcriptomic or proteomic validation was performed in this study. These findings suggest that PE-1 can colonize PE, initiate oxidative cleavage, and potentially assimilate breakdown products. This study provides new insights into the microbial degradation of polyolefins and identifies a promising bacterial candidate for plastic bioremediation.

## 1. Introduction

Plastic pollution has become a global environmental crisis. Polyethylene (PE) is one of the most produced and widely used materials among synthetic plastics, with an estimated annual production reaching 121.4 million tons by 2026 [1,2]. Due to its high crystallinity, non-polar aliphatic backbone, and extremely low surface energy, PE is highly chemically inert and resistant to biodegradation, allowing it to persist in natural environments [3]. As plastic waste continues to accumulate, especially in closed ecosystems such as landfills, PE gradually fragments into micro- and nanoplastics [4]. These particles are now widely detected in soil, water, and even food chains, posing potential risks to ecological safety and human health [4,5].

Conventional disposal methods such as incineration and landfilling are not only resource-intensive but also risk releasing toxic emissions or occupying large areas of land. Mechanical recycling faces challenges due to contamination, mixed compositions, and polymer degradation during use. Therefore, developing green and sustainable biodegradation technologies has emerged as a key research focus in recent years [1]. Although PE is considered one of the most recalcitrant plastics, several studies over the past decade have shown that certain microorganisms can attack its structure. For example, *Pseudomonas* sp., *Lysinibacillus* sp., and *Bacillus* sp. have been reported to grow on PE surfaces and cause measurable weight loss and functional group changes [6,7,8]. Notably, Yang et al. demonstrated that *Enterobacter asburiae* and *Bacillus* sp. isolated from the gut of *Galleria mellonella* could degrade low-density polyethylene (LDPE) within days, drawing significant scientific attention [9]. However, most of these studies rely on pre-treated PE, modified by UV, thermal oxidation, or ozone exposure, and evidence for direct degradation of untreated, commercial-grade PE remains limited [1].

More importantly, current research often stops at phenotypic observations, such as weight loss, surface erosion seen under scanning electron microscopy (SEM), or new functional groups detected by Fourier Transform infrared spectroscopy (FTIR) without mechanistic insights [1]. Which enzymes initiate oxidation? Are there specific metabolic pathways involved? From a chemical structure perspective, PE is essentially a long-chain n-alkane, and theoretically, it can be degraded via metabolic pathways similar to those involved in alkane degradation [10,11,12]. Under aerobic conditions, the initial oxidation of long-chain alkanes typically occurs at the terminal or subterminal positions. Enzymes such as cytochrome P450 monooxygenases, alkane hydroxylases (pAHs), and those associated with the β-oxidation pathway provide a theoretical foundation for understanding the potential biodegradation of PE [10,13]. Despite related reports, information on the structural properties of PE-degrading enzymes and the associated metabolic pathways remains relatively limited [1,10]. Urban municipal solid waste landfills represent unique ecosystems where microbial communities are chronically exposed to complex mixtures of plastic waste. Over time, this long-term selective pressure may favor the evolution of microbes capable of recognizing, colonizing, and potentially utilizing plastic components [14]. Thus, landfill environments serve as promising reservoirs for discovering novel and environmentally relevant degraders [15]. However, the microbial mechanisms underlying polyethylene degradation in these ecosystems remain poorly understood, particularly at the intersection of community-level enrichment and strain-level functional genomics.

Microbial degradation of polyethylene is generally hypothesized to involve a multi-enzymatic process. Given the inert nature of the C–C backbone, initial oxidation is often considered the rate-limiting step, mediated by oxidoreductases such as laccases, multicopper oxidases, or monooxygenases [10]. These enzymes introduce oxygen-containing functional groups (e.g., carbonyl, hydroxyl) into the polymer chain, reducing molecular weight and increasing hydrophilicity. Subsequently, hydrolases including esterases, lipases, and depolymerases may act on these oxidized regions to further cleave the polymer into assimilable oligomers [6,11]. While most depolymerases are characterized against polyesters [12], understanding the distribution and diversity of these functional genes in landfill-derived microbes is crucial for elucidating the molecular mechanisms of PE biodegradation.

The objectives of this study were to: (1) enrich and characterize a microbial consortium capable of degrading untreated, commercial-grade polyethylene using landfill soil as the inoculum source; (2) isolate and identify pure bacterial strains with high degradation activity from the enriched consortium; and (3) elucidate the genetic basis of PE degradation potential through whole-genome sequencing and functional annotation of the isolated strain. To achieve these objectives, soil samples were collected from a typical domestic waste landfill in China and subjected to a long-term enrichment strategy using food-grade PE as the sole carbon source. This approach successfully yielded a stable degrading consortium (designated PEH) and a highly active strain, *Bacillus cereus* PE-1. Multi-level physicochemical analyses, including weight loss measurement, SEM, and thermogravimetric analysis (TGA), were employed to confirm PE degradation. Furthermore, whole-genome sequencing and functional annotation were performed to identify gene clusters associated with depolymerization and to predict potential metabolic pathways underlying PE degradation. This study provides integrated phenotypic and genomic insights into microbial PE degradation, contributing to the development of biological strategies for plastic waste mitigation.

## 2. Materials and Methods

### 2.1. Sample Collection and Enrichment of PE-Degrading Microbial Consortia

The substrate used was commercial food-grade PE film and microplastics (Guanghong Polymer Materials Management Department (Dongguan, China)). According to the manufacturer’s specifications, the film has a density of 0.92 g/cm^3^ and a thickness of 0.2 μm. No additives or plasticizers were included. Soil samples were collected from three different locations within an active domestic waste landfill site in Beijing, Yantai and Suihua, China. Samples were taken from the top 10–20 cm layer where visible plastic debris was abundant. Soil samples from three locations were homogenized and pooled into a composite sample before inoculation into a single enrichment flask. To enrich for PE-utilizing microbes, 5 g of fresh soil was inoculated into 100 mL of mineral salt medium (MSM) containing (per liter): NaCl (0.5 g), K_2_HPO_4_ (1.0 g), KH_2_PO_4_ (0.5 g), NH_4_NO_3_ (1.0 g), MgSO_4_·7H_2_O (0.2 g), CaCl_2_ (0.01 g), FeSO_4_·7H_2_O (0.001 g), and trace element solution (1 mL). Trace elements were added as follows: CuSO_4_·5H_2_O (0.001 g), MnSO_4_·5H_2_O (0.001 g), ZnSO_4_·7H_2_O (0.001 g). A composite vitamin B stock solution (containing thiamine/B1, riboflavin/B2, niacin/B3, and pyridoxine/B6, each at 0.5 g/L) was prepared separately. One milliliter of this stock solution was added per liter of MSM. The vitamin solution was filter-sterilized (0.22 μm) and added after autoclaving to prevent thermal degradation. PE film pieces (1 × 1 cm^2^, previously washed with ethanol and dried) were added as the sole carbon source. Cultures were incubated at 30 °C with shaking at 150 rpm. Every 7 days, 10% (*v*/*v*) of the culture was transferred into fresh MSM supplemented with new PE films. After six cycles (~6 weeks), a stable degrading consortium designated PEH was obtained. The 6-week enrichment process was conducted as a screening step to isolate PE-utilizing microorganisms through serial transfers in MSM with PE film as the sole carbon source. Abiotic controls were not included during this screening phase. Following enrichment, a formal 30-day degradation assay was conducted with parallel abiotic controls to quantify degradation and distinguish biological effects from abiotic weathering. The composition of the PEH consortium was determined by high-throughput 16S rRNA gene amplicon sequencing targeting the V3–V4 regions on an Illumina MiSeq platform (Illumina, San Diego, CA, USA).

### 2.2. Isolation and Identification of Pure PE-Degrading Strains

Serial dilutions of the PEH consortium were plated onto nutrient agar supplemented with 0.1% yeast extract. Single colonies showing distinct morphologies were picked and purified. Isolates were then re-inoculated into PE-supplemented MSM to confirm degradation ability. A strain exhibiting consistent growth and visible PE surface alteration was selected for identification. Genomic DNA was extracted, and the 16S rRNA gene was amplified using universal primers 27F and 1492R. The PCR product was sequenced using Sanger sequencing (approximately 1430 bp). This sequence was used for phylogenetic analysis. Phylogenetic analysis was conducted using BLAST (v2.17.0) and MEGA X (v 10.2.6) software with the neighbor-joining method.

### 2.3. Degradation Assay and Analytical Characterization

Before inoculation, the bacterial cells were harvested by centrifugation and washed three times with sterile MSM to minimize the carryover of organic nutrients from the pre-culture media. While we acknowledge that a specific “MSM without PE” control was not included in this study, the rigorous washing protocol was designed to reduce this risk. To minimize the influence of residual biomass on measurements, we implemented a rigorous cleaning protocol before analysis. After the 30-day incubation, PE samples (film and microplastics) were: (1) rinsed three times with sterile distilled water to remove loose cells; (2) washed with 0.1% SDS solution for 10 min to detach biofilm; (3) sonicated for 5 min in sterile water; and (4) dried at 50 °C until reaching constant weight.

#### 2.3.1. Weight Loss Measurement

Pre-weighed sterile PE films (initial dry weight *W*_0_) were incubated with the PEH consortium or pure strain in 50 mL MSM for 30 days. Films were retrieved, rinsed thoroughly with distilled water to remove biomass, dried to constant weight, and re-weighed (*W_t_*). Control groups included sterile controls (autoclaved inoculum) and abiotic controls (no inoculum) [16]. The percentage weight loss was calculated using the following formula:

$$Weight\;Loss\;(\%)=\left(1-\frac{w_{t}}{w_{0}}\right)\times 100$$

All treatments, including abiotic controls, underwent identical cleaning and weighing procedures. Each experiment was performed in triplicate (*n* = 3), and results are presented as mean ± standard deviation.

#### 2.3.2. Surface Morphology Analysis

Scanning electron microscopy (SEM, Hitachi SU8010, Hitachi, Tokyo, Japan) was used to observe surface morphology changes. Samples were sputter-coated with gold prior to imaging. Detailed instrument parameters, sample preparation procedures, and imaging conditions are provided in the Supplementary Materials Section.

#### 2.3.3. Hydrophilicity Assessment

Contact angles were measured using a contact angle meter (Krüss DSA100, KRÜSS Scientific, Hamburg, Germany) with deionized water droplets (5 μL). At least five measurements per sample were averaged.

#### 2.3.4. Elemental Analysis

The dried microplastic samples were ground and then combusted in pure oxygen for thermal conductivity detection to determine the C and H content of the samples. The oxygen content was measured by pyrolyzing the samples at 1150 °C in a H_2_/He mixture, followed by reduction with carbon to convert it to CO, and then performing thermal conductivity detection [16].

#### 2.3.5. Thermal Stability Evaluation

Thermogravimetric analysis (TGA, TA Instruments Q500, Waters, Milford, MA, USA) was performed under nitrogen atmosphere (flow rate: 40 mL/min), heating from 30 °C to 600 °C at 10 °C/min. Initial decomposition temperature (IDT) was defined as the temperature at 5% weight loss.

### 2.4. Whole-Genome Sequencing and Bioinformatics Analysis

Genomic DNA of *Bacillus cereus*. PE-1 was extracted and subjected to paired-end sequencing (150 bp) using the Illumina NovaSeq 6000 platform (Illumina, San Diego, CA, USA). Raw reads were quality-filtered using Trimmomatic v0.39, followed by de novo assembly with SPAdes v3.15.0. Genome completeness and contamination were assessed using CheckM (v1.1.6). Annotation was performed through the NCBI PGAP and RAST platforms. The 16S rRNA gene sequence was also extracted from the assembled genome for confirmation and Average Nucleotide Identity (ANI) analysis.

Key predictions focused on genes encoding oxidoreductases, hydrolases, and known plastic-degrading enzymes such as depolymerase, laccase, and esterase. Multiple sequence alignment was performed using the MUSCLE (v5.3) (MUltiple Sequence Comparison by Log-Expectation) algorithm [17].

### 2.5. Nucleotide Sequence Accession Numbers

The genome sequence of *Bacillus cereus* PE-1 has been deposited in the GenBank database under the accession number PRJNA1399039.

### 2.6. Statistical Analysis

All experiments were conducted in triplicate. Data are presented as mean ± standard deviation. One-way ANOVA followed by Tukey’s test was used for statistical comparisons (*p* < 0.05 considered significant), implemented in SPSS v26.0.

## 3. Results

### 3.1. Isolation of the PE-Degrading Microbial Consortium PEH and the Strain PE-1

**Figure 1:**
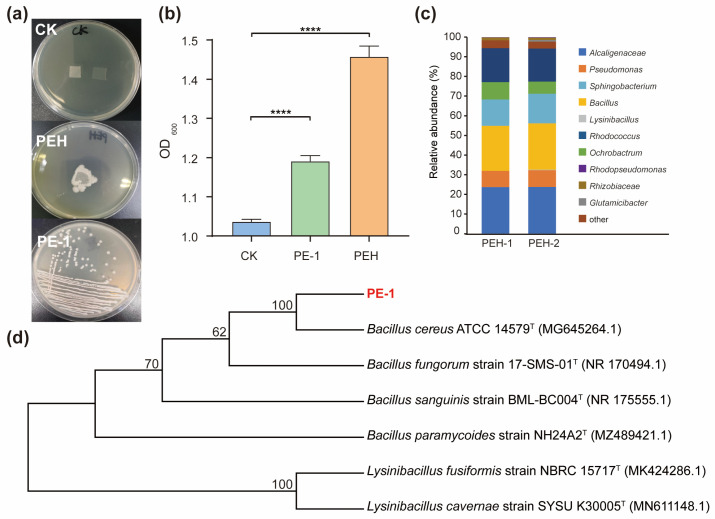
Isolation and characterization of the PE-degrading consortium PEH and strain *Bacillus cereus* PE-1. (**a**) Colony morphology of the microbial consortium PEH and pure strain PE-1 on MSM agar plates supplemented with PE film as the sole carbon source. (**b**) Growth dynamics of PEH and PE-1 in liquid MSM-PE cultures monitored by OD_600_ over 30 days; sterilized control (CK) shows no increase. (**c**) Microbial composition of the PEH consortium at the genus level based on 16S rRNA gene sequencing. (**d**) Phylogenetic tree showing the taxonomic position of strain PE-1, constructed using 16S rRNA gene sequences via the neighbor-joining method; bootstrap values (%) from 1000 replicates are shown at branch nodes. Statistical significance is indicated as follows: **** *p* < 0.0001.

In this study, a functional microbial consortium capable of utilizing PE as a sole carbon source was successfully enriched from landfill soil through long-term cultivation in MSM supplemented with PE film as the only carbon source. As shown in Figure 1a, visible colonies developed on MSM-PE plates, indicating that the consortium, named PEH, had adapted to oligotrophic conditions and gained the ability to use inert polymers for growth.

Through serial dilution plating and repeated streaking, a single bacterial strain capable of stable growth in MSM + PE was isolated and designated PE-1. The strain formed circular, creamy-white, smooth, and moist colonies on solid medium. OD_600_ monitoring revealed continuous growth of both the PEH consortium and strain PE-1 under PE-dependent conditions (Figure 1b). Initial OD6_00_ of the sterilized control (CK) was 1.04. After 30 d, OD6_00_ reached 1.19 for the PE-1 culture and 1.46 for the PEH consortium, both significantly higher than CK (*p* < 0.0001). While this suggests microbial growth in the presence of PE, we acknowledge that residual carryover cannot be fully excluded without a carbon-free control.

**Figure 2:**
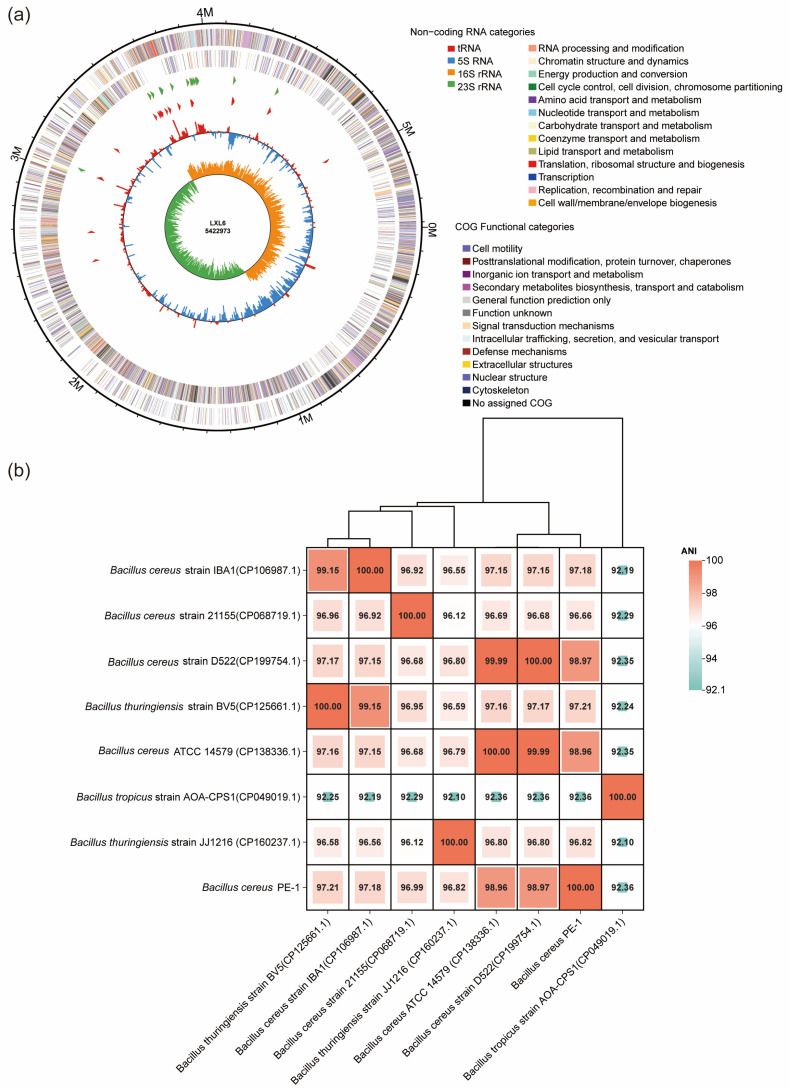
Genome features and average nucleotide identity (ANI) analysis of *Bacillus cereus* PE-1. (**a**) Circular representation of the complete genome of *B. cereus* PE-1, showing GC skew (outer), GC content (middle), and predicted coding sequences (inner). (**b**) ANI heatmap comparing *B. cereus* PE-1 with closely related type strains.

To characterize its composition, the PEH consortium was analyzed by high-throughput 16S rRNA gene sequencing. At the genus level (Figure 1c), PEH was dominated by *Alcaligenes* (23.88%), *Bacillus* (23.17%), *Rhodopseudomonas* (17.0%), *Sphingobacterium* (14.26%), and *Pseudomonas* (8.39%). For taxonomic identification of PE-1, a phylogenetic tree based on 16S rRNA gene sequences was constructed (Figure 1d), and whole-genome sequencing and assembly were performed (Figure 2a). Phylogenetic analysis showed that PE-1 clustered closely with *Bacillus cereus* ATCC 14579, sharing 99.8% sequence similarity, suggesting it belongs to *Bacillus*. Furthermore, average nucleotide identity (ANI) analysis based on the full genome revealed 98.96% identity with *B. cereus* ATCC 14579 (Figure 2b), supporting its classification as *Bacillus cereus*, here named *Bacillus cereus* PE-1.

### 3.2. Degradation Characterization

#### 3.2.1. Surface Erosion of PE by Consortium PEH and Strain PE-1

**Figure 3:**
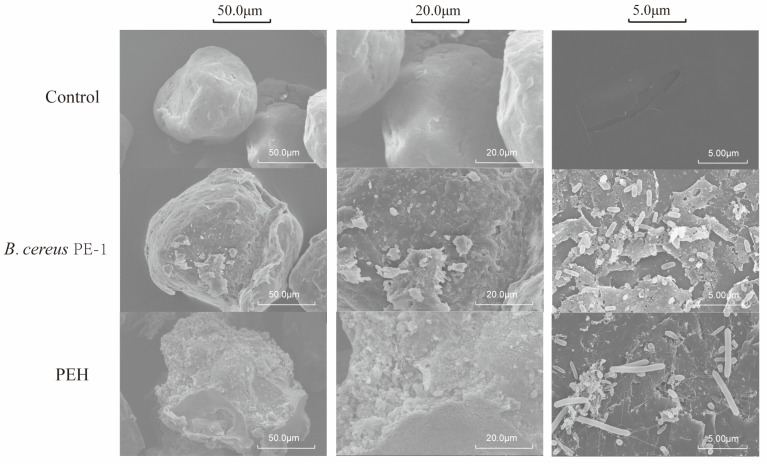
SEM images of PE microplastics after microbial treatment. Scale bars: 5 μm, 20 μm and 50 μm.

To evaluate the degradative activity of the PEH consortium and the pure strain PE-1, SEM was used to examine the surface morphology of PE microplastics after treatment (Figure 3). Untreated PE exhibited a smooth and compact surface without visible defects. In contrast, samples exposed to either PEH or *Bacillus cereus* PE-1 showed clear signs of structural damage, including deep cracks, flake-like exfoliation, pore formation, and local collapse.

#### 3.2.2. Weight Loss and Reduced Thermal Stability of PE

**Figure 4:**
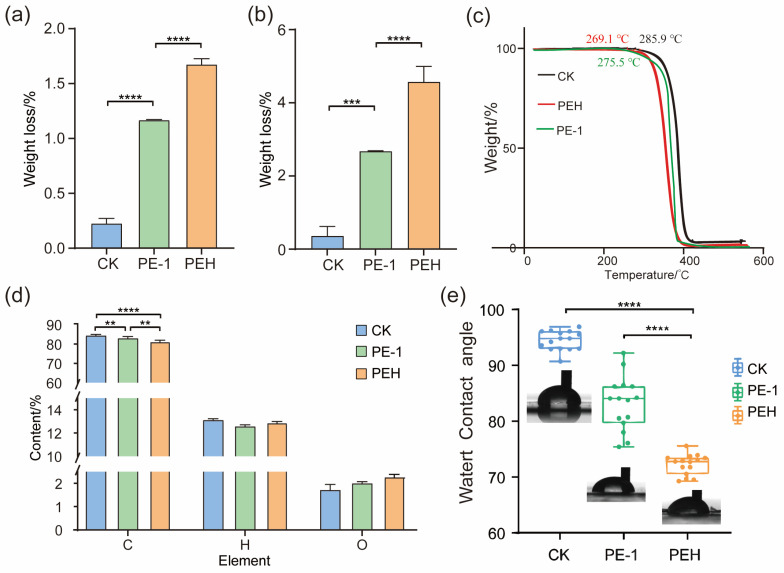
Physicochemical characterization of PE degradation. Weight loss of PE films (**a**) and microplastic particles (**b**) after 30 days of incubation with PEH, PE-1, or in non-inoculated control (CK); error bars represent standard deviation (*n* = 3); significant differences were determined by one-way ANOVA. (**c**) TGA curves showing reduced onset degradation temperature after microbial treatment. (**d**) Element analysis revealing increased oxygen content in treated samples. (**e**) Water contact angle indicating enhanced hydrophilicity following microbial colonization. Statistical significance is indicated as follows: ** *p* < 0.01, *** *p* < 0.001, **** *p* < 0.0001.

To further quantify degradation, weight loss of PE films and microplastic particles was measured after microbial treatment. After 30 days of incubation, the PEH consortium and strain *Bacillus cereus* PE-1 caused 1.64% and 1.17% weight loss in PE films, respectively (Figure 4a). These values were significantly higher than that of the non-inoculated control (CK: 0.22%, *p* < 0.0001). A similar trend was observed for microplastic particles (Figure 4b). In this case, the control group showed 0.36% weight loss, while PEH and PE-1 treatments resulted in 4.57% and 2.68% weight loss, respectively, both significantly exceeding the control (*p* < 0.001). TGA was conducted to assess changes in thermal stability. All samples exhibited a single-step and complete thermal decomposition process. The onset degradation temperature, the temperature at which 5% mass loss occurred, was 285.9 °C for the control and dropped to 269.1 °C for the PEH-treated sample (Figure 4c), representing a 5.88% reduction. IDT value for PE-1 treated PE is 275.5 °C, which also represents a significant decrease compared to the control (Figure 4c).

#### 3.2.3. Changes in Surface Chemical Properties: Oxidation and Increased Hydrophilicity

The elemental composition of PE before and after microbial treatment was analyzed (Figure 4d). Untreated PE consisted mainly of carbon (84.54%) and hydrogen (13.16%). After treatment with *Bacillus cereus* PE-1 and the PEH consortium, carbon content decreased to 83.12% and 81.40%, respectively, while hydrogen levels dropped slightly to 12.62% and 12.91%. Notably, oxygen was detected in both treated samples, with contents of 2.02% and 2.30% for PE-1 and PEH, respectively.

Consistent with these chemical changes, water contact angle measurements showed that pristine PE films were highly hydrophobic, with an initial contact angle of 95.5° ± 2.81° (Figure 4e). After 30 days of treatment, the contact angles decreased significantly to 72.3° ± 1.71° for PEH and 83.8° ± 6.85° for PE-1, representing reductions of 24.29% and 12.25%, respectively (*p* < 0.0001).

### 3.3. Genome of Bacillus cereus PE-1 Reveals Potential Genes Involved in PE Degradation

**Figure 5:**
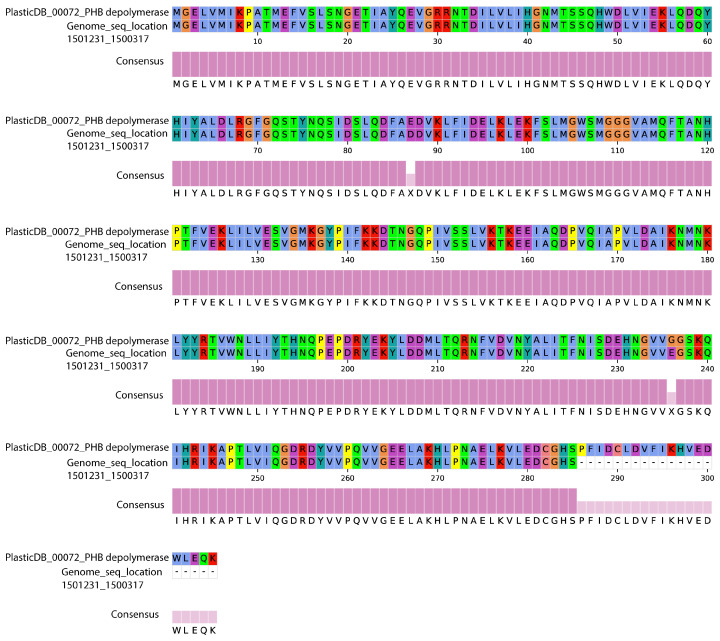
Protein sequence alignment of the PHB depolymerase in *Bacillus cereus* PE-1. Amino acid sequence alignment between the predicted depolymerase from PE-1 and homologs from *Bacillus thuringiensis*, showing high sequence similarity (99.34%).

**Figure 6:**
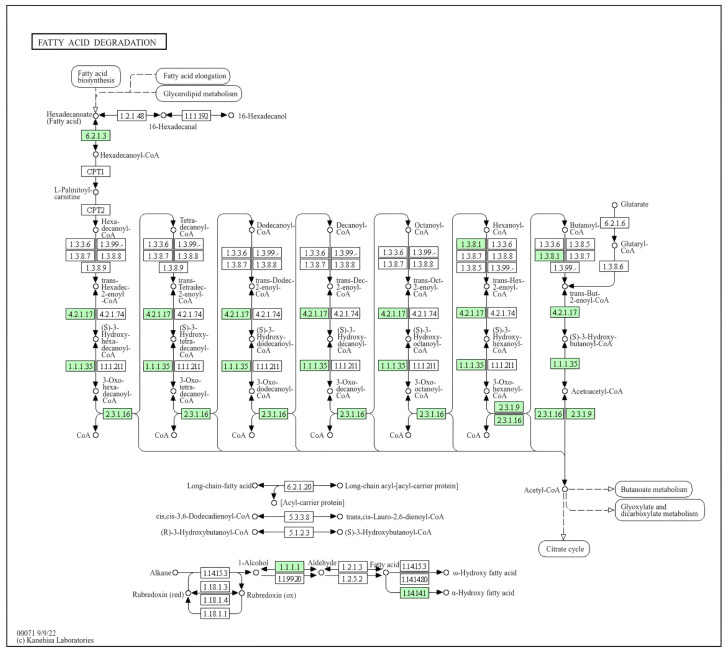
Schematic illustration of the β-oxidation pathway in *Bacillus cereus* PE-1. Highlighted in green are the enzymes annotated in *B. cereus* PE-1.

In this study, the efficient PE-degrading strain *Bacillus cereus* PE-1 was subjected to whole-genome sequencing and functional annotation. The assembled genome consists of a single circular chromosome of 5,422,972 bp with no plasmids, a GC content of 35.33%, and 5553 predicted protein-coding genes. KEGG-based annotation identified homologs of *epsA* and *epsB*, genes associated with extracellular polymeric substance (EPS) biosynthesis (Table 1). Based on PlasticDB annotation, one depolymerase gene in the PE-1 genome showed 99.34% similarity to a PHB depolymerase from *Bacillus thuringiensis* (Table 2 and Table S1). This enzyme consists of 285 amino acids (Figure 5). While not a direct PE degrader, this enzyme may facilitate the breakdown of oxidized intermediates. Its presence suggests a potential capacity for processing polyester-like structures that may emerge during advanced stages of PE oxidation. A putative laccase homolog gene was also identified, sharing 33% sequence similarity with a homolog from *Rhodococcus ruber* (Table 2 and Figure S1). Two copper oxidase genes were additionally detected, showing 30.84% and 34.58% similarity to known sequences (Table 2, Figures S2 and S3). While this sequence similarity is at the threshold of homology and does not confirm functional equivalence, the presence of conserved copper-binding motifs suggests potential oxidase activity. However, no enzymatic assays (e.g., ABTS or syringaldazine oxidation) were performed in this study, and this gene should be considered a structural homolog requiring functional validation. Importantly, genes related to the β-oxidation pathway were also identified in the PE-1 genome, including long-chain acyl-CoA synthetase (ACSL), acetyl-CoA C-acetyltransferase (ACAT), and alcohol dehydrogenase (adhE) (Figure 6 and Table S2).

## 4. Discussion

### 4.1. PE Degradation Efficiency and Surface Modification

Several *Bacillus* species have previously been reported to degrade synthetic plastics. For example, *Bacillus cereus* strain HM989919 was shown to degrade polylactic acid (PLA) and polyethylene terephthalate (PET) in heavy metal-contaminated soils [24]. Another study isolated a *B. cereus* strain from mangrove sediment and demonstrated its ability to degrade PE, PET, and polystyrene (PS) by 1.6%, 6.6%, and 7.4%, respectively, within 40 d [25]. Although some *B. cereus* strains are conditionally pathogenic, their high environmental resilience, spore-forming capacity, and ability to secrete diverse extracellular enzymes make them promising candidates for bioremediation applications [7,8]. Bacterial cells were clearly attached to the polymer surface and were observed extending along fissures into the material (Figure 3). This indicates that these microbes not only colonize the PE surface but also penetrate its physical matrix, leading to structural breakdown. Microbial colonization is the first critical step in PE biodegradation. Numerous studies have demonstrated that biofilm formation enhances interactions between bacteria and the hydrophobic PE surface [26]. For instance, biofilm-forming bacteria such as *Rhodococcus ruber* exhibit stronger adhesion and higher degradation efficiency toward LDPE compared to non-biofilm-forming strains [22]. Fungal hyphae can firmly attach to PE surfaces. They are known to adhere to nearly all types of solid materials, enabling stable microbial colonization [27]. Once attached, microorganisms can use PE or its oxidation products as a carbon source for sustained growth. The morphological changes observed here provide direct microscopic evidence of biological activity on PE. These features distinguish microbial degradation from abiotic processes such as physical aging or non-enzymatic oxidation.

These results of weight loss and TGA demonstrate that microbial degradation occurs across different physical forms of PE, including both films and microparticles (Figure 4b,c). While phenotypic degradation was observed, detailed polymer characterization (e.g., GPC for Mn/Mw) will be included in future mechanistic studies to further quantify chain scission. The higher weight loss observed in microplastic particles compared to films may be attributed to differences in surface area. Microplastics have a larger specific surface area, which facilitates greater bacterial attachment and enhances enzymatic access, thereby accelerating degradation. This finding aligns with previous reports showing faster degradation rates for microplastics than bulk polymer films under comparable conditions [28]. TGA decrease indicates structural damage to the PE chains, including disruption of crystalline regions and overall polymer loosening. As a result, the material becomes more susceptible to thermal breakdown. A lowered onset temperature is a key indicator of polymer depolymerization [29]. The leftward shift of the TGA curve provides strong evidence of main-chain scission induced by microbial activity, supporting the conclusion that biological processes contribute to the intrinsic deterioration of PE [30]. In addition, microbial activity has been shown to alter the elemental composition of plastics (Figure 4d). Liu et al. reported that after degradation by *Bacillus velezensis* C5, carbon content in PE significantly decreased while nitrogen content increased [31]. This suggests that the bacterium utilized PE as a carbon source while simultaneously secreting large amounts of proteins. In this study, carbon content also significantly declined in degraded PE compared to the control (CK), consistent with Liu et al., indicating that the degrading strains used PE or its breakdown products as a carbon source for growth [31]. Although nitrogen was not detected in our samples, this may be due to the removal of microbial cells and extracellular proteins during the washing steps prior to analysis. Liu et al. also observed an increase in oxygen-containing functional groups such as R–OH and C–O on PE surfaces after degradation, suggesting oxidative modification [32]. The appearance of oxygen in our results supports similar oxidation reactions occurring during microbial treatment. Surface hydrophobicity is closely related to the polarity of chemical groups and surface topography [27,28]. The observed reduction in contact angle reflects an increase in surface polarity, which enhances moisture retention, nutrient diffusion, and secondary microbial colonization. This creates a positive feedback loop that promotes sustained degradation over time. These findings collectively indicate that strain PE-1 likely secreted oxidoreductases, such as monooxygenases and peroxidases, that initiated auto-oxidation of the inert hydrocarbon chains. This process converted nonpolar PE into more polar compounds, altering its surface chemistry and creating conditions favorable for further microbial attack.

### 4.2. Genomic Insights into Proposed Degradation Mechanisms

The genome of *Bacillus cereus* PE-1 reveals potential genes involved in PE degradation (Table 1 and Table 2). EPS, composed of polysaccharides, proteins, nucleic acids, and lipids, forms the structural scaffold of biofilms and plays a key role in cell-surface adhesion, intercellular aggregation, and environmental protection [33]. Its presence is critical for successful microbial colonization of microplastics [34,35,36]. Additionally, a gene encoding PgaC, a transmembrane glycosyltransferase required for the synthesis of poly-β-1,6-N-acetyl-D-glucosamine (PNAG), was detected. PNAG is a conserved component of bacterial biofilm matrices across diverse species [37,38]. The presence of these genes suggests that PE-1 has the genetic potential to form stable surface-associated communities, which may enhance its ability to colonize inert materials such as PE. It should be emphasized that this study only confirms the genomic potential for EPS production. There is currently no experimental evidence, such as gene expression, protein detection, or phenotypic validation, to demonstrate that these genes are expressed during microplastic colonization. Therefore, while *epsA*, *epsB*, and *pgaC* provide a plausible molecular basis for surface attachment, their functional roles must be verified through future experiments, including gene knockout, EPS quantification, and confocal microscopy (Table 1). Enzymatic activity plays a central role in various steps of PE biodegradation [39]. Several putative plastic-degrading enzymes have been reported in public databases such as PlasticDB [40,41,42]. Laccases belong to the multicopper oxidase (MCO) superfamily and can reduce oxygen to water without generating harmful byproducts [43,44]. Previous studies have shown that such enzymes can initiate radical-mediated oxidation of tertiary carbon atoms along the PE chain, triggering auto-oxidation cascades and leading to main-chain scission [45,46]. The presence of putative laccase and copper oxidase homologs in PE-1 (Figure 5 and Table 2), combined with our earlier observation of increased oxygen content after degradation, supports their potential role in the initial oxidative attack on PE. Moreover, esterase and lipase genes were annotated (Table 2). Although traditionally known for hydrolyzing ester bonds, these enzymes may also act on oxidized end groups or labile “micro-ester”-like structures formed on PE after oxidation [47]. When PE is partially oxidized into ketones, aldehydes, or alcohols, local polarity increases, potentially forming transient polar domains susceptible to enzymatic hydrolysis [48]. This process could release low-molecular-weight organic compounds, facilitating deeper degradation. These genes of β-oxidation pathway suggest that once PE is oxidized and cleaved into short-chain fatty acids, they can be activated via acyl-CoA synthetase and enter the β-oxidation cycle (Figure 6 and Table S2) [49]. Through successive rounds of dehydrogenation, hydration, and thiolytic cleavage, these intermediates are converted into acetyl-CoA, which then enters the tricarboxylic acid (TCA) cycle for complete mineralization [50,51]. This metabolic framework not only explains how microbes might utilize PE-derived carbon but also provides strong theoretical support for true biodegradation beyond mere surface modification.

### 4.3. Proposed Pathway for PE Assimilation and Mineralization

Based on these results, a four-stage model for PE degradation by *Bacillus cereus* PE-1 is proposed: (1) colonization, (2) depolymerization, (3) assimilation, and (4) potential mineralization. In the first stage, colonization, PE-1 shows strong surface adaptability. The presence of *epsA*, *epsB*, and *pgaC* supports its capacity to produce EPS and PNAG, enabling stable biofilm formation on hydrophobic PE surfaces. Biofilm development enhances cell–cell cohesion and creates a localized environment enriched with extracellular enzymes, promoting sustained chemical attack. This trait parallels findings in *Rhodococcus ruber*, where robust biofilm formation correlates with enhanced LDPE degradation efficiency [22]. In the second stage, depolymerization, laccase and copper oxidases likely mediate the initial oxidation of PE. These multicopper oxidases may generate radicals that attack tertiary carbons, initiating auto-oxidation reactions. This leads to chain scission and the introduction of oxygen-containing functional groups such as hydroxyl and carbonyl moieties, consistent with observed increases in O-content and reduced contact angles. Furthermore, the presence of esterase, lipase, and PHB depolymerase suggests the potential hydrolysis of oxidized terminal or pseudo-polyester regions, generating soluble small molecules like alcohols and fatty acids [52]. The genome of PE-1 contains a gene with 99.34% similarity to a PHB depolymerase from *Bacillus* thuringiensis. While this enzyme primarily targets ester bonds in polyhydroxybutyrate, it may potentially act on oxidized PE fragments containing ester-like functional groups formed after initial oxidative attack by laccases or monooxygenases. However, we acknowledge that this connection remains speculative without direct biochemical validation. Therefore, this enzyme should be considered circumstantially relevant as part of a downstream processing toolkit rather than a primary initiator of PE chain scission. While changes in Mn/Mw were not directly measured, SEM images showing surface erosion and TGA indicating reduced thermal stability indirectly support polymer chain breakdown. In the third stage, assimilation, short-chain fatty acids generated during fragmentation must be transported into the cell. Although specific metabolites such as dodecanoic acid or suberic acid were not yet detected, the presence of a complete set of β-oxidation core enzymes strongly implies that PE-1 can activate and metabolize exogenous fatty acids. This hypothesis is further supported by the continuous increase in OD_600_, indicating active growth using PE or its derivatives as a carbon source. While OD_600_ monitoring and SEM imaging suggest the presence of intact biomass after 30 days, we acknowledge that specific viable cell counts (CFU/mL) were not determined at the endpoint. Given the spore-forming capability of *Bacillus cereus*, the observed biomass likely includes both vegetative cells and endospores. Future studies will incorporate time course viability assays to precisely quantify metabolic activity throughout the degradation process. Finally, in the potential mineralization stage, acetyl-CoA enters the TCA cycle and is ultimately oxidized to CO_2_ and H_2_O [49]. In contrast, Stages 3 and 4 are inferred from genomic annotations of β-oxidation and TCA cycle pathways. While these genetic markers suggest metabolic potential, direct validation through isotopic tracing or metabolite detection was not performed in this study. Therefore, this model should be regarded as a hypothetical framework guiding future mechanistic investigations. Future studies will employ isotopic tracing to quantify the extent of carbon mineralization.

In summary, this study integrates phenotypic evidence, including weight loss, surface erosion, and chemical modification, with genomic insights into functional enzyme systems and metabolic pathways. Together, these data indicate that *Bacillus cereus* PE-1 is not limited to superficial modifications but possesses a comprehensive suite of genetic tools to initiate multi-level degradation of PE. Future work should focus on functional validation of key enzymes, tracking of metabolic intermediates, and dissection of regulatory networks to transition from “genetic potential” to “functional realization,” thereby advancing the application of this strain in plastic bioremediation.

## 5. Conclusions

This study successfully isolated a PE-degrading microbial consortium, designated PEH, and a pure bacterial strain, *Bacillus cereus* PE-1. Both were shown to colonize PE surfaces and induce structural and chemical changes in the polymer. Genomic analysis of PE-1 revealed a suite of functional genes associated with oxidation, hydrolysis, and fatty acid metabolism, suggesting its potential to attach to PE, modify its surface, and utilize degradation products as carbon sources. These findings demonstrate that *Bacillus cereus* PE-1 possesses the genetic foundation for multi-step PE biodegradation. This strain represents a novel microbial resource for studying polyolefin degradation mechanisms and provides a promising basis for developing bio-based strategies to mitigate plastic pollution.

## Figures and Tables

**Table 1:** Genes involved in EPS biosynthesis were annotated in the KEGG database.

Strain PE-1 Genomic Location	KO/Gene_ID	KEGG_Gene_Name
LXL6003539	K19420	*epsA*
LXL6005358	K19420	*epsA*
LXL6003538	K00903	*epsB*
LXL6003540	K00903	*epsB*
LXL6001639	K11936	*pgaC*

**Table 2:** Genes related to the degradation of microplastic were annotated in PlasticDB.

Genome_Location	Enzyme Type	Identify (%)	Coverage (%)	E-Value	Plastic Type	Reference
1501231_1500317	PHB depolymerase	99.344	100	0	PHB\PHA	[18]
779388_780410	Esterase	53.846	82	1.77 × 10^−114^	PBAT	[19]
779388_780527	Lipase	53.197	95	3.20 × 10^−128^	PBAT	[20]
540813_540013	Protease	47.212	99	1.56 × 10^−56^	PLA	[21]
55346_55645	Laccase	33	72	2.55 × 10^−10^	LDPE	[22]
54635_56170	Copper oxidase	30.84	91	3.57 × 10^−66^	PE	[23]
54743_55138	Copper oxidase	34.586	73	6.55 × 10^−20^	PE	[23]

## Data Availability

The original contributions presented in this study are included in the article. Further inquiries can be directed to the corresponding author.

## Supplementary Materials

The following supporting information can be downloaded at https://www.mdpi.com/article/10.3390/polym18060695/s1. Figure S1: Protein sequence alignment of the Laccases in *Bacillus cereus* PE-1; Figure S2: Protein sequence alignment of the copper oxidase in *Bacillus cereus* PE-1; Figure S3: Protein sequence alignment of the copper oxidase in *Bacillus cereus* PE-1; Table S1: The genes related to the degradation of PE annotated in the PlasticDB database; Table S2: The genes related to the pathway of fatty acid degradation in KEGG annotation [18,19,20,21,22,23,40,41].

## Abbreviations

The following abbreviations are used in this manuscript:

PE	Polyethylene
SEM	Scanning electron microscopy
TGA	Thermogravimetric analysis
FTIR	Fourier transform infrared spectroscopy
