# Supplementary material for: Phenotypic and Genomic Characterization of Polyethylene-Degrading Bacillus cereus PE-1 Enriched from Landfill Microbial Consortium
Source: Polymers (Basel). 2026 Mar 12;18(6):695. doi: 10.3390/polym18060695 (PMC13030748; doi:10.3390/polym18060695)
Supplement: Supplementary file 1 [file polymers-18-00695-s001.zip › polymers-4159587-supplementary.pdf]

Phenotypic and Genomic Characterization of Polyethylene-Degrading *Bacillus cereus* PE-1 Enriched from Landfill Microbial Consortium

Weijun Wang, Shunyu Yao, Zhimin Liu and Xiaolu Liu *

School of Chemistry and Biological Engineering, University of Science and Technology Beijing, 30 Xueyuan Road, Haidian District, Beijing 100083, China; wangweijun@ustb.edu.cn (W.W.); yaoshunyu3d21@163.com (S.Y.); liuzhimin_1@126.com (Z.L.)

* Correspondence: xiaoluliu@ustb.edu.cn

Academic Editors: Dan Huang, Mingqiang Zhu and Yanyan Dong

Received: 2 February 2026
Revised: 28 February 2026
Accepted: 11 March 2026
Published: 12 March 2026

Copyright: © 2026 by the authors. Submitted for possible open access publication under the terms and conditions of the Creative Commons Attribution (CC BY) license.

Materials and Methods

Scanning electron microscopy (SEM). SEM was used to confirm the proliferation of the degrading bacteria on the PE surface and the degradation of the PE. Prior to SEM observation, the bacteria on the PE films and MPs were not washed to observe the adhered bacteria. PE films and MPs were first washed three times with phosphate buffer solution to remove the medium and fixed with 2.5% glutaraldehyde-containing phosphate buffer solution (0.1 M, pH = 7.0) for 4 h. Then, they were fixed in 1% OsO4 in phosphate buffer for 1 h, followed by dehydration in a gradient from 30% to 100% in absolute ethanol for 30 min, and finally dried in a critical point dryer. The dried samples were placed in a vacuum evaporator and sprayed with gold for 25 s to improve the conductivity of the samples. The samples were placed under a scanning electron microscope. The accelerating voltage was set at 2 KV. The shooting angle was 360 °.

Table S1 The genes related to the degradation of PE were annotated in PlasticDB database

Genome_seq_location	PlasticDB ID	Enzyme type	Identify （%）	Coverage （%）	E_value	Bit_score	Degradation species	Plastic type	Reference
779316_780530	00049	PLA depolymerase	46.262	96	2.02e-106	343	Uncultured bacterium	PBS\PBSA\PCL\PES\PHB\PLA\PHA	[41]
779388_780533	00050	PLA depolymerase	48.608	91	1.45e-105	340	Uncultured bacterium	PBS\PBSA\PCL\PES\PHB\PLA\PHA	[41]

1501231_1500317	00072	PHB depolymerase	99.344	100	0	629	Bacillus thuringiensis	PHB\PHA	[18]
779388_780410	00077	Esterase	53.846	82	1.77e-114	366	Clostridium botulinum	PBAT	[19]
779388_780542	00078	Esterase	42.965	91	1.02e-98	321	Clostridium botulinum	PBAT	[19]
779388_780527	00080	Lipase	53.197	95	3.20e-128	405	Pelosinus fermentans	PBAT	[20]
540813_540013	00152	Protease	47.212	99	1.56e-56	192	Lederbergia lenta	PLA	[21]
152594_153349	00152	Protease	41.27	99	7.87e-37	136	Lederbergia lenta	PLA	[21]
2545210_2545467	00152	Protease	45.349	99	2.69e-14	70.5	Lederbergia lenta	PLA	[21]
2544262_2544582	00152	Protease	47.273	99	2.01e-13	67.8	Lederbergia lenta	PLA	[21]
540813_540013	00153	Protease	46.84	99	2.05e-63	213	Lederbergia lenta	PLA	[21]
152594_153349	00153	Protease	40.873	99	4.28e-43	154	Lederbergia lenta	PLA	[21]
2544262_2544582	00153	Protease	47.273	99	3.21e-18	82	Lederbergia lenta	PLA	[21]
2545210_2545467	00153	Protease	45.349	99	2.87e-14	70.1	Lederbergia lenta	PLA	[21]
540900_540013	00154	Protease	43.377	77	1.26e-54	191	Bacillus	PLA	[21]

							licheniformis		
152597_153451	00154	Protease	40.702	77	2.29e-40	150	Bacillus licheniformis	PLA	[21]
2544253_2544582	00154	Protease	41.129	77	3.86e-15	75.1	Bacillus licheniformis	PLA	[21]
1125019_1125348	00209	Oxidoreductase	40.909	99	1.27e-19	89.7	Bacillus velezensis	PU\PBAT	[40]
55346-55645	00174	Laccase	33	72	2.55e-10	60.5	Rhodococcus ruber	LDPE	[22]
54635_56170	00222	Copper oxidase	30.84	91	3.57e-66	230	Rhodococcus opacus	PE	[23]
54743_55138	00223	Copper oxidase	34.586	73	6.55E-20	89	Rhodococcus opacus	PE	[23]

Table S2 The genes related to the pathway of fatty acid degradation in KEGG annotation

Gene_location	Entry	Pathway	Description
1416987_1420184	K14338	ko00071	cypD_E, CYP102A, CYP505; cytochrome P450 / NADPH-cytochrome P450 reductase [EC:1.14.14.1 1.6.2.4]

441456_442493	K13953	ko00071	adhP; alcohol dehydrogenase, propanol-preferring [EC:1.1.1.1]
2700711_2701499	K13767	ko00071	fadB; enoyl-CoA hydratase [EC:4.2.1.17]
3153693_3156074	K07516	ko00071	fadN; 3-hydroxyacyl-CoA dehydrogenase [EC:1.1.1.35]
2557081_2559684	K04072	ko00071	adhE; acetaldehyde dehydrogenase / alcohol dehydrogenase [EC:1.2.1.10 1.1.1.1]
2702193_2703926	K01897	ko00071	ACSL, fadD; long-chain acyl-CoA synthetase [EC:6.2.1.3]
4762111_4763625	K01897	ko00071	ACSL, fadD; long-chain acyl-CoA synthetase [EC:6.2.1.3]
3152499_3153671	K00632	ko00071	fadA, fadI; acetyl-CoA acyltransferase [EC:2.3.1.16]
1800718_1801809	K00626	ko00071	ACAT, atoB; acetyl-CoA C-acetyltransferase [EC:2.3.1.9]
2237135_2238310	K00626	ko00071	ACAT, atoB; acetyl-CoA C-acetyltransferase [EC:2.3.1.9]
3478353_3479636	K00626	ko00071	ACAT, atoB; acetyl-CoA C-acetyltransferase [EC:2.3.1.9]
710859_712004	K00248	ko00071	ACADS, bcd; butyryl-CoA dehydrogenase [EC:1.3.8.1]
3475087_3476226	K00248	ko00071	ACADS, bcd; butyryl-CoA dehydrogenase [EC:1.3.8.1]

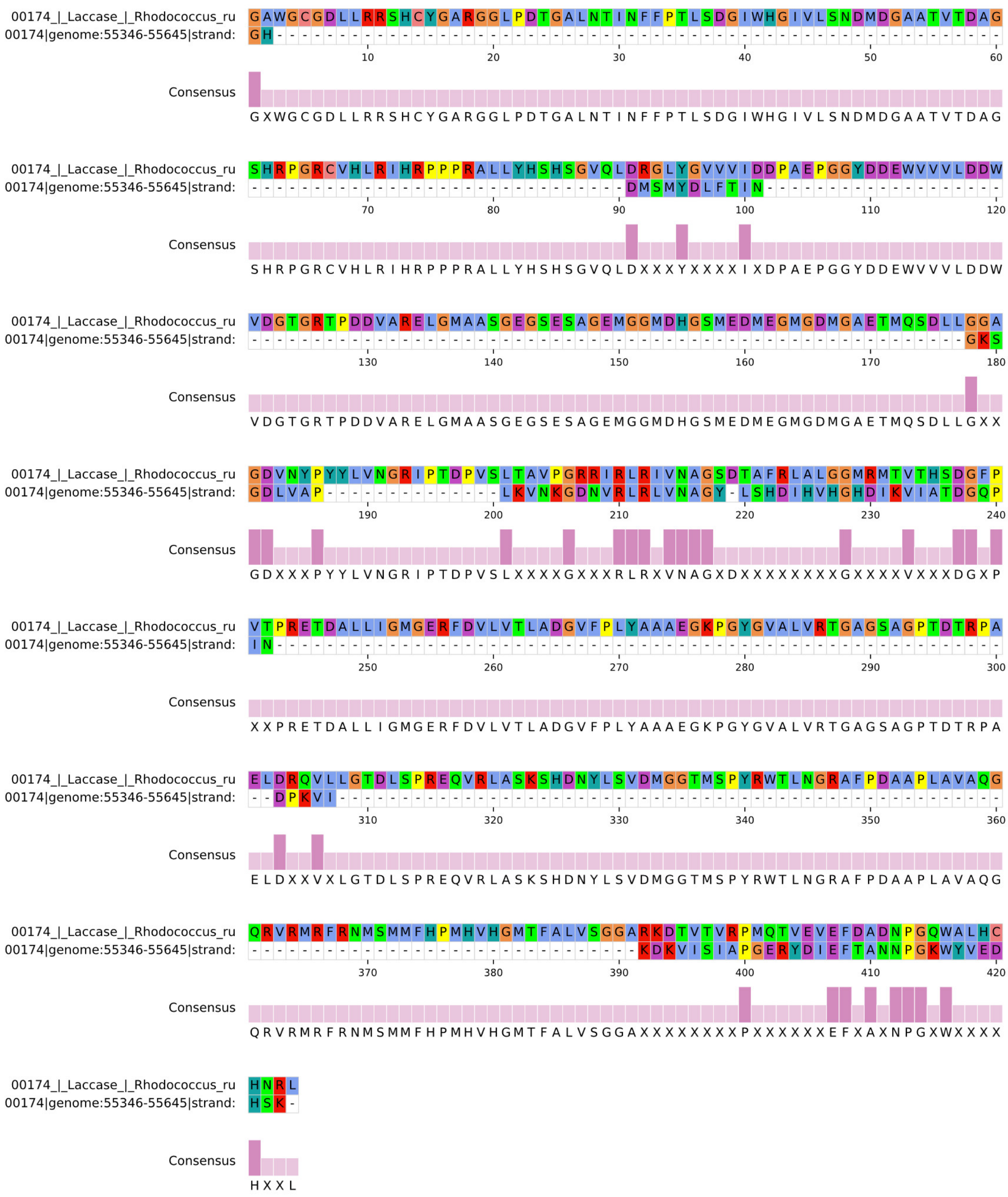

Figure S1 Protein sequence alignment of the Laccases in *Bacillus cereus* PE-1.

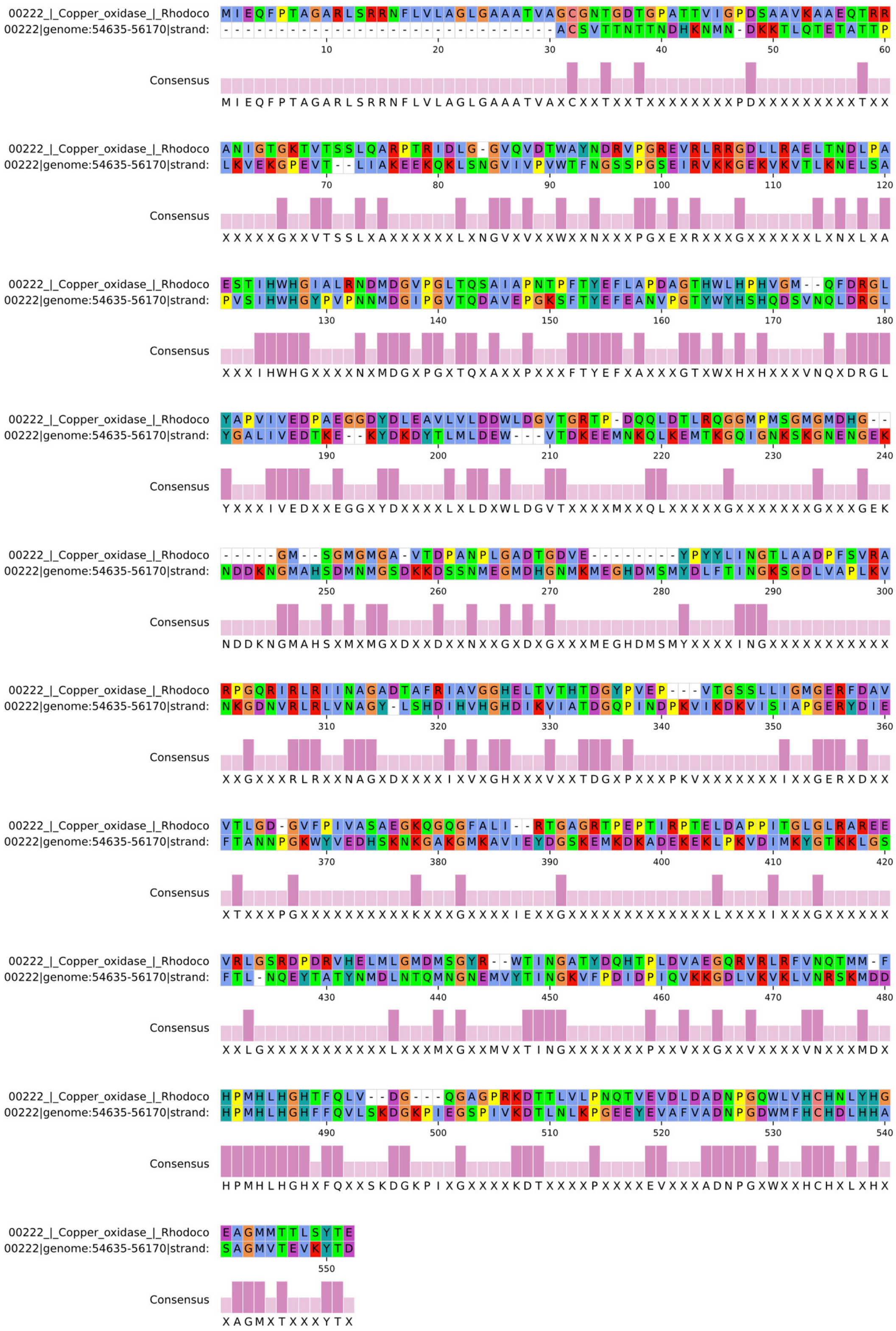

Figure S2 Protein sequence alignment of the copper oxidase in *Bacillus cereus* PE-1.

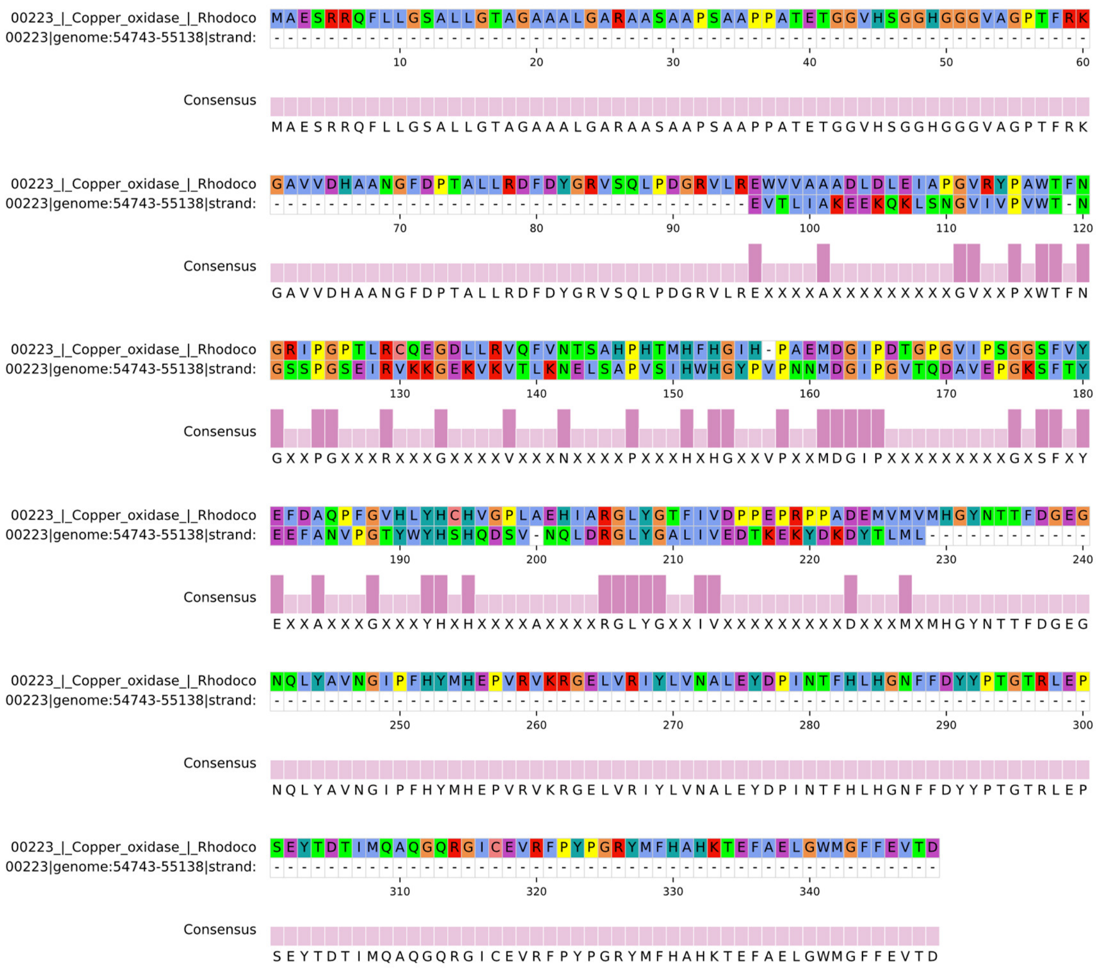

Figure S3 Protein sequence alignment of the copper oxidase in *Bacillus cereus* PE-1.

Gene sequence of degradation-related enzymes in *Bacillus cereus* PE-1

>00049|genome:779316-780530|strand:plus|identity:46.262%|evalue:2.02E-106|coverage:96%|

ATATGTTTTTTTGCATTAGTGACCGTATGTTCATTCGCAGCGAAAATATCATACGCTGAA
GAGAGGCAACAAAATAATTATCCTATCATATTGGTGAATGGATTTGCTGGATGGGGTAGA
GAAGAAATGCTTGGCGTAAAATATTGGGGTGGTGTTCATGATATACAGGAAGATTTGAAA
CGAAATGGTTATACGGTACATACCGCAGCTGTTGGACCTGTTTCTAGTAACTGGGATCGT
GCATGTGAATTGTATGCACAAATTAGCGGTGGGACAGTAGACTATGGTGCAGTCCATGCT
GAGAAACATGGACATAATCGTTTTGGCAGAACTTATAGTGGTTTTGCGCCGAATTGGAGT
GAAACAAATAAAGTTCATTTAGTTGGACATAGCATGGGTGGACAAACGATT

AGAACATTA
GTACAGCTATTAAAAGAAGGTAGTTTTGAAGAAAAGAATTATGTGAAAAAT
CACCCAGAC
ACCAAAATATCACCACTATTTGAAGGCGGAAAATCATATGTTCATAGTGTTA
CAACATTA
GCAACCCCTCACAACGGTACAACACTTGCGGATGGTAGTCTTCTACTGCCG
TTCGTTAAA
GATTTACTCATTACAACTGCAAGCTTTGGGGGAAACAATAATTTATCATTATA
TGATTTT
AAATTGGATCAATGGGGTATAAAGAAGAATGCTGGAGAGTCCTTTTTCCAA
TATAGTAAT
CGTATTCTAAATAGTTCACTTTGGAAAAATACAAAAGATATAAGTCAATGGG
ATTTAAGT
ACAGATGGTGCAAAGGAGTTAAATAATTGGGTAAAGACGCAACCAGATGT
GTATTATTTA
TCATATAGTGGACATGCATCACAAGCAGCACCTATAACAGGTTTACATTTGC
CTCATATA
ACGATGAATAAAGTGTTAATGGGAAATGCATTTTTCTTAGGTTCTTATGCGA
GATATGAA
GAAAACCGTCCGCTAGTTGATACTTCTTGGTGGCAAAATGATGGTGTAGTA
AATACAAAT
TCTATGATTGCACCATCTTCTAATGTTACTGTAAATAATAATGAGTCATTACA
GATTGGA
AAATGGAATCATATCGAAACGAAAGCGAATTGGGACCATCTCGACATGGTA
GGGTTAAGT
GTTTCAGACTCGTTAGGTTTTTCTAGCATTCAAGAGTTTTATAGAACAATTG
CTGAAAAG
CTATCACGTTTACCG
**>00050|genome:779388-780533|strand:plus|identity:48.608%|evalue:1.45E-105|c
overage:91%|**
AATAATTATCCTATCATATTGGTGAATGGATTTGCTGGATGGGGTAGAGAAG
AAATGCTT
GGCGTAAAATATTGGGGTGGTGTTCATGATATACAGGAAGATTTGAAACGA
AATGGTTAT
ACGGTACATACCGCAGCTGTTGGACCTGTTTCTAGTAACTGGGATCGTGCAT
GTGAATTG
TATGCACAAATTAGCGGTGGGACAGTAGACTATGGTGCAGTCCATGCTGAG
AAACATGGA
CATAATCGTTTTGGCAGAACTTATAGTGGTTTTGCGCCGAATTGGAGTGAAA
CAAATAAA
GTTCATTTAGTTGGACATAGCATGGGTGGACAAACGATTAGAACATTAGTAC
AGCTATTA
AAAGAAGGTAGTTTTGAAGAAAAGAATTATGTGAAAAATCACCCAGACAC
CAAAATATCA

CCACTATTTGAAGGCGGAAAATCATATGTTCATAGTGTTACAACATTAGCAA
CCCCTCAC
AACGGTACAACACTTGCGGATGGTAGTCTTCTACTGCCGTTCGTTAAAGAT
TTACTCATT
ACAACTGCAAGCTTTGGGGGAAACAATAATTTATCATTATATGATTTTAAATT
GGATCAA
TGGGGTATAAAGAAGAATGCTGGAGAGTCCTTTTTCCAATATAGTAATCGTA
TTCTAAAT
AGTTCACTTTGGAAAAATACAAAAGATATAAGTCAATGGGATTTAAGTACA
GATGGTGCA
AAGGAGTTAAATAATTGGGTAAAGACGCAACCAGATGTGTATTATTTATCAT
ATAGTGGA
CATGCATCACAAGCAGCACCTATAACAGGTTTACATTTGCCTCATATAACGA
TGAATAAA
GTGTTAATGGGAAATGCATTTTTCTTAGGTTCTTATGCGAGATATGAAGAAA
ACCGTCCG
CTAGTTGATACTTCTTGGTGGCAAAATGATGGTGTAGTAAATACAAATTCTA
TGATTGCA
CCATCTTCTAATGTTACTGTAAATAATAATGAGTCATTACAGATTGGAAAATG
GAATCAT
ATCGAAACGAAAGCGAATTGGGACCATCTCGACATGGTAGGGTTAAGTGTT
TCAGACTCG
TTAGGTTTTTCTAGCATTCAAGAGTTTTATAGAACAATTGCTGAAAAGCTAT
CACGTTTA
CCGAAA

>00072|genome:1501231-1500317|strand:minus|identity:99.344%|evalue:0|coverage:100%|direction

TTTCTGTTCTAACCAATCCTCTACATGTTTTATAAATACATCTAAACAATCAA
TAAACGG
CGAGTGTCCGCAATCTTCTAATACCTTCAACTCTGCATTTGGCAAATGTTTC
GCTAATTC
CTCACCGACTACTTGCGGTACGACATAATCTCTATCACCTTGTATGACGAGT
GTTGGCGC
TTTAATACGATGAATTTGTTTACTTCCCTCTACAACCCCATTATGTTCATCTG
AAATATT
AAATGTAATGAGCGCATAATTCACATCTACGAAATTACGTTGCGTTAACATA
TCATCTAA
ATACTTTTCATAACGATCCGGTTCAGGTTGATTATGTGTATATATTAATAGATT
CCATAC
TGTACGGTAATATAGTTTGTTCATATTTTTATCGCATCTAATACTGGAGCGAT
TTGTAC
TGGATCTTGCGCAATTTCTTCTTTCGTCTTTACTAAACTTGATACAATCGGCT
GCCCATT
CGTATCTTTTTTAAAGATTGGGTATCCTTTCATTCCTACTGATTCTACTAAAA

TTAACTT
TTCTACAAAAGTTGGGTGATTCGCTGTAAATTGCATCGCAACGCCACCACC
CATTGACCA
GCCCATTAATGAAAATTTCTCTAGCTTTAACTCGTCAATAAATAATTTTACAT
CGTCTGC
AAAGTCTTGTAATGAATCTATCGACTGATTATACGTTGATTGTCCAAACCCT
CTTAAATC
AAGAGCGTAAATATGGTATTGATCTTGCAACTTTTCAATAACTAAATCCCAA
TGTTGTGA
CGATGTCATGTTCCCGTGAATGAGTACAAGAATATCTGTATTTCGCCTTCCA
ACTTCCTG
ATATGCAATCGTTTCTCCGTTCGATAGTGAAACAAACTCCATTGTTGCAGGC
TTAATCAT
CACAAGTTCCCCCAT
>00077|genome:779388-780410|strand:plus|identity:53.846%|evalue:1.77E-114|coverage:82%|
AATAATTATCCTATCATATTGGTGAATGGATTTGCTGGATGGGGTAGAGAAG
AAATGCTT
GGCGTAAAATATTGGGGTGGTGTTCATGATATACAGGAAGATTTGAAACGA
AATGGTTAT
ACGGTACATACCGCAGCTGTTGGACCTGTTTCTAGTAACTGGGATCGTGCAT
GTGAATTG
TATGCACAAATTAGCGGTGGGACAGTAGACTATGGTGCAGTCCATGCTGAG
AAACATGGA
CATAATCGTTTTGGCAGAACTTATAGTGGTTTTGCGCCGAATTGGAGTGAAA
CAAATAAA
GTTCATTTAGTTGGACATAGCATGGGTGGACAAACGATTAGAACATTAGTAC
AGCTATTA
AAAGAAGGTAGTTTTGAAGAAAAGAATTATGTGAAAAATCACCCAGACAC
CAAAATATCA
CCACTATTTGAAGGCGGAAAATCATATGTTCATAGTGTTACAACATTAGCAA
CCCCTCAC
AACGGTACAACACTTGCGGATGGTAGTCTTCTACTGCCGTTCGTTAAAGAT
TTACTCATT
ACAACTGCAAGCTTTGGGGGAAACAATAATTTATCATTATATGATTTTAAATT
GGATCAA
TGGGGTATAAAGAAGAATGCTGGAGAGTCCTTTTTCCAATATAGTAATCGTA
TTCTAAAT
AGTTCACTTTGGAAAAATACAAAAGATATAAGTCAATGGGATTTAAGTACA
GATGGTGCA
AAGGAGTTAAATAATTGGGTAAAGACGCAACCAGATGTGTATTATTTATCAT
ATAGTGGA
CATGCATCACAAGCAGCACCTATAACAGGTTTACATTTGCCTCATATAACGA
TGAATAAA

GTGTTAATGGGAAATGCATTTTTCTTAGGTTCTTATGCGAGATATGAAGAAA
ACCGTCCG
CTAGTTGATACTTCTTGGTGGCAAAATGATGGTGTAGTAAATACAAATTCTA
TGATTGCA
CCATCTTCTAATGTTACTGTAAATAATAATGAGTCATTACAGATTGGAAAATG
GAATCAT
ATC
>00078|genome:779388-780542|strand:plus|identity:42.965%|evalue:1.02E-98|coverage:91%|
AATAATTATCCTATCATATTGGTGAATGGATTTGCTGGATGGGGTAGAGAAG
AAATGCTT
GGCGTAAAATATTGGGGTGGTGTTCATGATATACAGGAAGATTTGAAACGA
AATGGTTAT
ACGGTACATACCGCAGCTGTTGGACCTGTTTCTAGTAACTGGGATCGTGCAT
GTGAATTG
TATGCACAAATTAGCGGTGGGACAGTAGACTATGGTGCAGTCCATGCTGAG
AAACATGGA
CATAATCGTTTTGGCAGAACTTATAGTGGTTTTGCGCCGAATTGGAGTGAAA
CAAATAAA
GTTCATTTAGTTGGACATAGCATGGGTGGACAAACGATTAGAACATTAGTAC
AGCTATTA
AAAGAAGGTAGTTTTGAAGAAAAGAATTATGTGAAAAATCACCCAGACAC
CAAAATATCA
CCACTATTTGAAGGCGGAAAATCATATGTTCATAGTGTTACAACATTAGCAA
CCCCTCAC
AACGGTACAACACTTGCGGATGGTAGTCTTCTACTGCCGTTCGTTAAAGAT
TTACTCATT
ACAACTGCAAGCTTTGGGGGAAACAATAATTTATCATTATATGATTTTAAATT
GGATCAA
TGGGGTATAAAGAAGAATGCTGGAGAGTCCTTTTTCCAATATAGTAATCGTA
TTCTAAAT
AGTTCACTTTGGAAAAATACAAAAGATATAAGTCAATGGGATTTAAGTACA
GATGGTGCA
AAGGAGTTAAATAATTGGGTAAAGACGCAACCAGATGTGTATTATTTATCAT
ATAGTGGA
CATGCATCACAAGCAGCACCTATAACAGGTTTACATTTGCCTCATATAACGA
TGAATAAA
GTGTTAATGGGAAATGCATTTTTCTTAGGTTCTTATGCGAGATATGAAGAAA
ACCGTCCG
CTAGTTGATACTTCTTGGTGGCAAAATGATGGTGTAGTAAATACAAATTCTA
TGATTGCA
CCATCTTCTAATGTTACTGTAAATAATAATGAGTCATTACAGATTGGAAAATG
GAATCAT
ATCGAAACGAAAGCGAATTGGGACCATCTCGACATGGTAGGGTTAAGTGTT

TCAGACTCG
TTAGGTTTTTCTAGCATTCAAGAGTTTTATAGAACAATTGCTGAAAAGCTAT
CACGTTTA
CCGAAATAGGTAATA
>00080|genome:779388-780527|strand:plus|identity:53.197%|evalue:3.2E-128|coverage:95%|
AATAATTATCCTATCATATTGGTGAATGGATTTGCTGGATGGGGTAGAGAAG
AAATGCTT
GGCGTAAAATATTGGGGTGGTGTTCATGATATACAGGAAGATTTGAAACGA
AATGGTTAT
ACGGTACATACCGCAGCTGTTGGACCTGTTTCTAGTAACTGGGATCGTGCAT
GTGAATTG
TATGCACAAATTAGCGGTGGGACAGTAGACTATGGTGCAGTCCATGCTGAG
AAACATGGA
CATAATCGTTTTGGCAGAACTTATAGTGGTTTTGCGCCGAATTGGAGTGAAA
CAAATAAA
GTTCATTTAGTTGGACATAGCATGGGTGGACAAACGATTAGAACATTAGTAC
AGCTATTA
AAAGAAGGTAGTTTTGAAGAAAAGAATTATGTGAAAAATCACCCAGACAC
CAAAATATCA
CCACTATTTGAAGGCGGAAAATCATATGTTCATAGTGTTACAACATTAGCAA
CCCCTCAC
AACGGTACAACACTTGCGGATGGTAGTCTTCTACTGCCGTTCGTTAAAGAT
TTACTCATT
ACAACTGCAAGCTTTGGGGGAAACAATAATTTATCATTATATGATTTTAAATT
GGATCAA
TGGGGTATAAAGAAGAATGCTGGAGAGTCCTTTTTCCAATATAGTAATCGTA
TTCTAAAT
AGTTCACTTTGGAAAAATACAAAAGATATAAGTCAATGGGATTTAAGTACA
GATGGTGCA
AAGGAGTTAAATAATTGGGTAAAGACGCAACCAGATGTGTATTATTTATCAT
ATAGTGGA
CATGCATCACAAGCAGCACCTATAACAGGTTTACATTTGCCTCATATAACGA
TGAATAAA
GTGTTAATGGGAAATGCATTTTTCTTAGGTTCTTATGCGAGATATGAAGAAA
ACCGTCCG
CTAGTTGATACTTCTTGGTGGCAAAATGATGGTGTAGTAAATACAAATTCTA
TGATTGCA
CCATCTTCTAATGTTACTGTAAATAATAATGAGTCATTACAGATTGGAAAATG
GAATCAT
ATCGAAACGAAAGCGAATTGGGACCATCTCGACATGGTAGGGTTAAGTGTT
TCAGACTCG
TTAGGTTTTTCTAGCATTCAAGAGTTTTATAGAACAATTGCTGAAAAGCTAT
CACGTTTA

>00152|genome:152594-153349|strand:plus|identity:41.27%|evalue:7.87E-37|coverage:99%|

GAAATTCCACCAGGTGTACAACTGATTCACGCTCCGCAAGTATGGGGAAAG
AGTGTGAAG
GGGCAAGATGTAGTCGTTGCCGTATTAGATACAGGGTGTGATACTAATCATA
TAGATTTA
AAAGATCGTATTATCGGCGGAAGAAATTTCACAAAAGATTATGAAGCTGAT
CCAAATGTG
TATCTTGATAATAACGGACATGGTACTCATGTCGCGGGGACGATTGCAGCAA
CTGAAAAT
GGTGTCGGTGTATTAGGAGTTGCACCACTTGCTAAAATGTTAGTATTAAAAG
TTTTAGCG
GGAGATGGTTCTGGAAGTTACGAGCAAATTATTGAGGCAATTCATTATGCTG
TAAATTGG
AGAGGGCCGAATCAAGAGAAAGTGAGAATTATTTCAATGTCACTTGGTGGT
CCGCAAGAT
GTTCCAGAATTACATGAAGCAATTCAAAATGCGGTAAAGCAGGATGTTCTC
GTCGTATGT
GCTGCTGGGAATAATGGAGATTGTAATGATAATACAGAGGAACTAGATTACC
CAGGTGCT
TACTCGGAAGTAATTGAAGTCGGCGCTGTCAATTTAGAACGGAAAATCACA
TGTTTTAGT
AATTCAAATCAAGAAATTGATCTAGTAGCGCCAGGTGATGAAATATTATCTA
CGTATCCA
GATGGGAAATATGCAGTGTTAAGTGGAACTTCCATGGCGACGCCGCATGTT
GCTGGAGCG
CTTGCCTTGCTCATTAAACAGTGTGAGCAAGAATAC

>00152|genome:2545210-2545467|strand:plus|identity:45.349%|evalue:2.69E-14|coverage:99%

GATGTTGTTGCACCTGGAGTACAAATTACTAGTACAGTACCGCGAGGCGGC
TATGAATCG
CATAACGGAACAAGTATGGCTGCTCCGCAAGTAGCTGGAGCGGTTGCCCTC
TTGCGTCAA
ATGCATCCTGATTGGACGACAGAACAATTGAAAGCGGCTCTTGCTAACAAT
GCAAAAACA
TTACATGATGTCAATGAAAATACATACCCTGTTATGGCACAAGGATCAGGTT
TAATTAAC
ATTCCGAAAGCAGCTCAA

>00152|genome:2544262-2544582|strand:plus|identity:47.273%|evalue:2.01E-13|coverage:99%|

AAACCGCTTGATGGAAAAGGCATGAAAGTAGCCATTATCGACTCTGGCGTA
GACTATACA
CACCCTGACCTAAAGGCAAATTATATCGGTGGATATGACACGGTTGATGAA
GATAACGAT

CCAATGGATGGTAACGTACATGGTACTCATGTAGCTGGAATTATTGCGGGTA
ACGGAAAA
ATTAAAGGCGTTGCTCCAAACGCTTCTATTCTAGCCTATCGTGTAATGAATG
ACGGTGGA
ACTGGTACAACAGATGATATTATCCAAGGAATTGAGCGAGCAATTCAAGAT
GGTGCGGAT
GTGTTAAACCTCTCCCTTGGG
>00152|genome:3038408-3038689|strand:plus|identity:39.362%|evalue:5.36E-11|coverage:99%|
GATATTACAGCGCCTGGTGTCGCTATCCTTTCAACAGTTCCTGAATATATTAA
CGACCCG
CAAGAGGGTGAAAACTACGCTGTTTCCTATGAGCGTATGCAAGGAACATCT
ATGGCTTCT
CCTCATATCGCTGGCGTTGCTGCTCTAATTTTACAAGAACACCCAGACTACT
CTCCGTTC
GATGTAAAAGCGTCTCTTATGAACACAGCTAATGATTTAAAAGAAAAATATT
CCGTATAT
GAAGTTGGAGCTGGGCGAGTAGATGCTTACAACGCCGTTCGT
>00152|genome:3037250-3037765|strand:plus|identity:34.302%|evalue:2.02E-10|coverage:99%|
CAAAAACTAACAGATAGCATCCCGCAAATTGGAGTAGATAAGTTACATAGT
GAGGGCATT
ACCGGAAAAGGAATTAAAGTTGGTGTATTAGATACAGGTATTGATTACAATC
ATCCTGAC
TTAAAAGACGTATATAAAGGCTATCGCGCGAAACCTGGTGAGGACTCTAGC
AAAGTAGAC
CTAAACTCAGTAAAGGGTTGGGACTTTATTAATAATGATGCAGATCCAATGG
AAACTACT
TATTCAGAATGGCAACAATCTGGTGCTCCTGAATTTGATAACCGTGGTTCTT
CCTTCTAC
ACAGCCCACGGCACTCACGTAGCAGGTATCGTTTCTGCCCAAAAGAAGAA
CCAATCTGAT
TCAGCAGTAAAAGGTGTTGCACCTGACATTGAACTGTATAACTATCGTGTAC
TCGGTCCA
TACGGAAGTGGAGATAGTTCAGGTATTATCGCTGCAATTGATAAATCCATTT
CTGACGGT
ATGAACGTTATTAACTTATCGTTAGGTGATGATAGC
>00153|genome:540813-540013|strand:minus|identity:46.84%|evalue:2.05E-63|coverage:99%|
TTGTACAGCCTTATATGCATTGACTCTACCGTTTTTCCAGTACGTACCTGTAC
CACTAAC
TTTATCAGAAGTTGACTCAATAATTTGGCGAATTTGTGTATTACTATATCCTT
GATTTGC
TAAAAGAGCAGCGACTCCTGCAACATGAGGTGTTGCCATAGATGTACCACT

TAATGATTG
ATACGTGCTTCCTTTATATGTTGAATATATATTTGAACCTGGTGCTGCAACAT
CTACCCA
GCTACCATAAGTAGAGAATGAAGATTTTCTATCTGATTGATCTGTAGAACCA
ACTGCAAT
TACTTCGCTGTAATAAGCAGGGTAATTAGCTTTTGTATTTCCAGCATTTCCAG
CAGCTGC
AACTATAACAGAGCCTTTATTCCATGCATATTGAACGGCTTGTTGTAATGCA
GTACCACC
ATTTGGAGCTCCTAAACTTAAACTAATTACTTTTGCACCCGAATCAGCAGCT
TCTCGAAT
ACCTTGCGCTACAGCATCAAGAGTACCACTTCCTTGATTATCTAATACGCGG
ACAGCATA
AATTGAAGTTTGTGGGGCAACACCAGCAATTCCGACGCTGTTATTCGTAAG
TGCTCCAGT
AATTCCAGCGCAATGTGTACCATGACCATTACCATCATCAGATGTATTGTCG
TTATCAAC
ATAATCATGCCCGTAAATTACTTTCGAAGCCAGATCAGGGTGTGAGCCTTGA
ACTCCTGT
ATCAATAATAGCTACTTTTACACCAGGATCACTTCGTTGGCTATCCCAAGCT
TGTGGAGC
TTGAATCTTTTGTAATCCATA
>00153|genome:152594-153349|strand:plus|identity:40.873%|evalue:4.28E-43|coverage:99%|
GAAATTCCACCAGGTGTACAACTGATTCACGCTCCGCAAGTATGGGGAAAG
AGTGTGAAG
GGGCAAGATGTAGTCGTTGCCGTATTAGATACAGGGTGTGATACTAATCATA
TAGATTTA
AAAGATCGTATTATCGGCGGAAGAAATTTCACAAAAGATTATGAAGCTGAT
CCAAATGTG
TATCTTGATAATAACGGACATGGTACTCATGTCGCGGGGACGATTGCAGCAA
CTGAAAAT
GGTGTCGGTGTATTAGGAGTTGCACCACTTGCTAAAATGTTAGTATTAAAAG
TTTTAGCG
GGAGATGGTTCTGGAAGTTACGAGCAAATTATTGAGGCAATTCATTATGCTG
TAAATTGG
AGAGGGCCGAATCAAGAGAAAGTGAGAATTATTTCAATGTCACTTGGTGGT
CCGCAAGAT
GTTCCAGAATTACATGAAGCAATTCAAAATGCGGTAAAGCAGGATGTTCTC
GTCGTATGT
GCTGCTGGGAATAATGGAGATTGTAATGATAATACAGAGGAACTAGATTACC
CAGGTGCT
TACTCGGAAGTAATTGAAGTCGGCGCTGTCAATTTAGAACGGAAAATCACA
TGTTTTAGT

AATTCAAATCAAGAAATTGATCTAGTAGCGCCAGGTGATGAAATATTATCTA
CGTATCCA
GATGGGAAATATGCAGTGTTAAGTGGAACTTCCATGGCGACGCCGCATGTT
GCTGGAGCG
CTTGCCTTGCTCATTAAACAGTGTGAGCAAGAATAC
>00153|genome:2544262-2544582|strand:plus|identity:47.273%|evalue:3.21E-18|coverage:99%|
AAACCGCTTGATGGAAAAGGCATGAAAGTAGCCATTATCGACTCTGGCGTA
GACTATACA
CACCCTGACCTAAAGGCAAATTATATCGGTGGATATGACACGGTTGATGAA
GATAACGAT
CCAATGGATGGTAACGTACATGGTACTCATGTAGCTGGAATTATTGCGGGTA
ACGGAAAA
ATTAAAGGCGTTGCTCCAAACGCTTCTATTCTAGCCTATCGTGTAATGAATG
ACGGTGGA
ACTGGTACAACAGATGATATTATCCAAGGAATTGAGCGAGCAATTCAAGAT
GGTGCGGAT
GTGTTAAACCTCTCCCTTGGG
>00153|genome:3037250-3037765|strand:plus|identity:33.721%|evalue:1.16E-15|coverage:99%|
CAAAAACTAACAGATAGCATCCCGCAAATTGGAGTAGATAAGTTACATAGT
GAGGGCATT
ACCGGAAAAGGAATTAAAGTTGGTGTATTAGATACAGGTATTGATTACAATC
ATCCTGAC
TTAAAAGACGTATATAAAGGCTATCGCGCGAAACCTGGTGAGGACTCTAGC
AAAGTAGAC
CTAAACTCAGTAAAGGGTTGGGACTTTATTAATAATGATGCAGATCCAATGG
AAACTACT
TATTCAGAATGGCAACAATCTGGTGCTCCTGAATTTGATAACCGTGGTTCTT
CCTTCTAC
ACAGCCCACGGCACTCACGTAGCAGGTATCGTTTCTGCCCAAAAGAAGAA
CCAATCTGAT
TCAGCAGTAAAAGGTGTTGCACCTGACATTGAACTGTATAACTATCGTGTAC
TCGGTCCA
TACGGAAGTGGAGATAGTTCAGGTATTATCGCTGCAATTGATAAATCCATTT
CTGACGGT
ATGAACGTTATTAACTTATCGTTAGGTGATGATAGC
>00153|genome:2545210-2545467|strand:plus|identity:45.349%|evalue:2.87E-14|coverage:99%|
GATGTTGTTGCACCTGGAGTACAAATTACTAGTACAGTACCGCGAGGCGGC
TATGAATCG
CATAACGGAACAAGTATGGCTGCTCCGCAAGTAGCTGGAGCGGTTGCCCTC
TTGCGTCAA
ATGCATCCTGATTGGACGACAGAACAATTGAAAGCGGCTCTTGCTAACAAT

GCAAAAACA
TTACATGATGTCAATGAAAATACATACCCTGTTATGGCACAAGGATCAGGTT
TAATTAAC
ATTCCGAAAGCAGCTCAA
>00153|genome:3038408-3038689|strand:plus|identity:39.362%|evalue:6.21E-11|coverage:99%|
GATATTACAGCGCCTGGTGTCGCTATCCTTTCAACAGTTCCTGAATATATTAA
CGACCCG
CAAGAGGGTGAAAACTACGCTGTTTCCTATGAGCGTATGCAAGGAACATCT
ATGGCTTCT
CCTCATATCGCTGGCGTTGCTGCTCTAATTTTACAAGAACACCCAGACTACT
CTCCGTTC
GATGTAAAAGCGTCTCTTATGAACACAGCTAATGATTTAAAAGAAAAATATT
CCGTATAT
GAAGTTGGAGCTGGGCGAGTAGATGCTTACAACGCCGTTCGT
>00154|genome:540900-540013|strand:minus|identity:43.377%|evalue:1.26E-54|coverage:77%|
TTGTACAGCCTTATATGCATTGACTCTACCGTTTTTCCAGTACGTACCTGTAC
CACTAAC
TTTATCAGAAGTTGACTCAATAATTTGGCGAATTTGTGTATTACTATATCCTT
GATTTGC
TAAAAGAGCAGCGACTCCTGCAACATGAGGTGTTGCCATAGATGTACCACT
TAATGATTG
ATACGTGCTTCCTTTATATGTTGAATATATATTTGAACCTGGTGCTGCAACAT
CTACCCA
GCTACCATAAGTAGAGAATGAAGATTTTCTATCTGATTGATCTGTAGAACCA
ACTGCAAT
TACTTCGCTGTAATAAGCAGGGTAATTAGCTTTTGTATTTCCAGCATTTCCAG
CAGCTGC
AACTATAACAGAGCCTTTATTCCATGCATATTGAACGGCTTGTTGTAATGCA
GTACCACC
ATTTGGAGCTCCTAAACTTAAACTAATTACTTTTGCACCCGAATCAGCAGCT
TCTCGAAT
ACCTTGCGCTACAGCATCAAGAGTACCACTTCCTTGATTATCTAATACGCGG
ACAGCATA
AATTGAAGTTTGTGGGGCAACACCAGCAATTCCGACGCTGTTATTCGTAAG
TGCTCCAGT
AATTCCAGCGCAATGTGTACCATGACCATTACCATCATCAGATGTATTGTCG
TTATCAAC
ATAATCATGCCCGTAAATTACTTTCGAAGCCAGATCAGGGTGTGAGCCTTGA
ACTCCTGT
ATCAATAATAGCTACTTTTACACCAGGATCACTTCGTTGGCTATCCCAAGCT
TGTGGAGC
TTGAATCTTTTGTAATCCATATTGATTATTAAAATATGGGTCGTTTGGAGTCC

AAAAGGC
GTGAACATAATAATTTGGTTCTGCATATTCCACATCTGGATTATTTTT
>00154|genome:152597-153451|strand:plus|identity:40.702%|evalue:2.29E-40|coverage:77%
ATTCCACCAGGTGTACAACTGATTCACGCTCCGCAAGTATGGGGAAAGAGT
GTGAAGGGG
CAAGATGTAGTCGTTGCCGTATTAGATACAGGGTGTGATACTAATCATATAGA
TTTAAAA
GATCGTATTATCGGCGGAAGAAATTTCACAAAAGATTATGAAGCTGATCCA
AATGTGTAT
CTTGATAATAACGGACATGGTACTCATGTCGCGGGGACGATTGCAGCAACT
GAAAATGGT
GTCGGTGTATTAGGAGTTGCACCACTTGCTAAAATGTTAGTATTAAAAGTTT
TAGCGGGA
GATGGTTCTGGAAGTTACGAGCAAATTATTGAGGCAATTCATTATGCTGTAA
ATTGGAGA
GGGCCGAATCAAGAGAAAGTGAGAATTATTTCAATGTCACTTGGTGGTCCG
CAAGATGTT
CCAGAATTACATGAAGCAATTCAAAATGCGGTAAAGCAGGATGTTCTCGTC
GTATGTGCT
GCTGGGAATAATGGAGATTGTAATGATAATACAGAGGAACTAGATTACCCA
GGTGCTTAC
TCGGAAGTAATTGAAGTCGGCGCTGTCAATTTAGAACGGAAAATCACATGT
TTTAGTAAT
TCAAATCAAGAAATTGATCTAGTAGCGCCAGGTGATGAAATATTATCTACGT
ATCCAGAT
GGGAAATATGCAGTGTTAAGTGGAACTTCCATGGCGACGCCGCATGTTGCT
GGAGCGCTT
GCCTTGCTCATTAAACAGTGTGAGCAAGAATACGGTCGAAAGTTATCAGAG
CCGGAAATA
TATGCACAACTTATTAAAAGAACTGTACCTTTAGGGTATGAACGTACATCCG
AAGGGAAT
GGATTATTAGATTTA
>00154|genome:2544253-2544582|strand:plus|identity:41.129%|evalue:3.86E-15|coverage:77%
CCATTTGGCAAACCGCTTGATGGAAAAGGCATGAAAGTAGCCATTATCGAC
TCTGGCGTA
GACTATACACACCCTGACCTAAAGGCAAATTATATCGGTGGATATGACACGG
TTGATGAA
GATAACGATCCAATGGATGGTAACGTACATGGTACTCATGTAGCTGGAATTA
TTGCGGGT
AACGGAAAAATTAAAGGCGTTGCTCCAAACGCTTCTATTCTAGCCTATCGT
GTAATGAAT
GACGGTGGAACTGGTACAACAGATGATATTATCCAAGGAATTGAGCGAGCA

ATTCAAGAT
GGTGCGGATGTGTTAAACCTCTCCCTTGGG
>00154|genome:3037250-3037765|strand:plus|identity:34.884%|evalue:3.47E-14|coverage:77%
CAAAAACTAACAGATAGCATCCCGCAAATTGGAGTAGATAAGTTACATAGT
GAGGGCATT
ACCGGAAAAGGAATTAAAGTTGGTGTATTAGATACAGGTATTGATTACAATC
ATCCTGAC
TTAAAAGACGTATATAAAGGCTATCGCGCGAAACCTGGTGAGGACTCTAGC
AAAGTAGAC
CTAAACTCAGTAAAGGGTTGGGACTTTATTAATAATGATGCAGATCCAATGG
AAACTACT
TATTCAGAATGGCAACAATCTGGTGCTCCTGAATTTGATAACCGTGGTTCTT
CCTTCTAC
ACAGCCCACGGCACTCACGTAGCAGGTATCGTTTCTGCCCAAAAGAAGAA
CCAATCTGAT
TCAGCAGTAAAAGGTGTTGCACCTGACATTGAACTGTATAACTATCGTGTAC
TCGGTCCA
TACGGAAGTGGAGATAGTTCAGGTATTATCGCTGCAATTGATAAATCCATTT
CTGACGGT
ATGAACGTTATTAACTTATCGTTAGGTGATGATAGC
>00154|genome:2545123-2545467|strand:plus|identity:36.522%|evalue:4.64E-11|coverage:77%
ATAAAAATTGGACAACCAAAGCAAACAGAACTTATCGGTAACTTTAGTTCA
AGAGGACCA
TCACAAGGAAGTTGGCTAATAAAGCCTGATGTTGTTGCACCTGGAGTACAA
ATTACTAGT
ACAGTACCGCGAGGCGGCTATGAATCGCATAACGGAACAAGTATGGCTGCT
CCGCAAGTA
GCTGGAGCGGTTGCCCTCTTGCGTCAAATGCATCCTGATTGGACGACAGAA
CAATTGAAA
GCGGCTCTTGCTAACAATGCAAAAACATTACATGATGTCAATGAAAATACAT
ACCCTGTT
ATGGCACAAGGATCAGGTTTAATTAACATTCCGAAAGCAGCTCAA
>00154|genome:3038357-3038638|strand:plus|identity:37.234%|evalue:1.73E-09|coverage:77%
GCAGACTTTAGCTCACGCGGTCCTGTTACAGCAAATGATGATATTAAGCCTG
ATATTACA
GCGCCTGGTGTCGCTATCCTTTCAACAGTTCCTGAATATATTAACGACCCGC
AAGAGGGT
GAAAACTACGCTGTTTCCTATGAGCGTATGCAAGGAACATCTATGGCTTCTC
CTCATATC
GCTGGCGTTGCTGCTCTAATTTTACAAGAACACCCAGACTACTCTCCGTTC
GATGTAAAA

GCGTCTCTTATGAACACAGCTAATGATTTAAAAGAAAAATAT
>00174|genome:55364-56122|strand:plus|identity:29.344%|evalue:3.71E-24|coverage:72%|
TACGACTTATTCACAATCAATGGAAAAAGCGGTGATTTAGTAGCGCCATTAAAAGTGAAT
AAGGGAGATAACGTTCGTCTTAGACTCGTCAATGCTGGTTATCTATCACATGATATACAT
GTTCATGGCCATGATATAAAAGTAATTGCGACAGATGGTCAACCAATAAACGATCCAAAA
GTTATAAAGGATAAAGTAATTTCAATCGCACCGGGTGAACGTTATGATATTGAATTTACT
GCTAATAATCCTGGGAAATGGTATGTTGAAGACCATTCGAAAAATAAAGGTGCAAAAGGA
ATGAAAGCTGTTATTGAGTATGATGGCAGCAAAGAGATGAAAGATAAAGCAGACGAAAAA
GAAAAATTACCGAAAGTAGATATAATGAAATATGGTACTAAAAAATTAGGTAGTTTCACA
TTAAATCAAGAGTATACTGCCACATATAATATGGACTTAAATACGCAAATGAATGGAAAT
GAAATGGTATATACAATTAACGGAAAGGTATTTCCGGATATTGACCCAATTCAAGTAAAA
AAGGGTGATTTAGTAAAAGTGAAGTTGGTAAATCGTTCTAAAATGGATGATCACCCAATG
CATTTACACGGACACTTTTTCCAGGTGTTGAGTAAAGATGGAAAACCGATAGAAGGTTCT
CCAATTGTGAAAGATACATTGAACTTAAAACCGGGAGAAGAATATGAAGTAGCCTTTGTA
GCAGATAATCCGGGCGATTGGATGTTCCACTGTCATGAT
>00174|genome:55346-55645|strand:plus|identity:33%|evalue:2.55E-10|coverage:72%|
GGTCATGATATGAGTATGTACGACTTATTCACAATCAATGGAAAAAGCGGTGATTTAGTA
GCGCCATTAAAAGTGAATAAGGGAGATAACGTTCGTCTTAGACTCGTCAATGCTGGTTAT
CTATCACATGATATACATGTTCATGGCCATGATATAAAAGTAATTGCGACAGATGGTCAA
CCAATAAACGATCCAAAAGTTATAAAGGATAAAGTAATTTCAATCGCACCGGGTGAACGT
TATGATATTGAATTTACTGCTAATAATCCTGGGAAATGGTATGTTGAAGACCATTCGAAA
>00174|genome:54911-55108|strand:plus|identity:40.299%|evalue:7.97E-07|coverage:72%|
TGGCATGGATATCCTGTCCCAAATAACATGGATGGAATTCCAGGCGTGACACAAGATGCA

GTTGAACCAGGAAAAAGTTTCACTTACGAATTTGAAGCGAACGTACCAGG
AACGTACTGG
TATCACTCGCATCAAGATTCTGTAAATCAATTAGATAGAGGTTTGTATGGAG
CGCTCATT
GTAGAAGATACAAAGGAA
>00209|genome:4043200-4041878|strand:minus|identity:39.198%|evalue:1.2E-94|coverage:99%
AGGAGGAATACTTTGTTGGAAATGAAAAACATCACAAGGATCATACATAGT
TTTTACTTT
ACGTAATCTGTGAAAGTTAGAGCCATAATAACTAGTTTGCCAATTTTTAATG
TCGATATC
GGGCCAGTTCACATAGTCACCTAGTGTATAAGGATCTAAGCTTTCTCGTAAA
CCTTTAAC
CCAGCGTATATTTCGATTTTCTTCATCATCGCATTTCCAAGAGGTAATGTATT
CTTGAGC
GATAATTGCTTTACGATGAAAATAAGCCGTTTCATTCGGCGAAATATTTTCA
ACAGCACC
TATGAGCGATTGATGCCAAATACTAGCATCTTTATTTGGTGCATGAGAAAGA
AAGGATTG
CATGATTTGAATGCCTTTAAGGGGAATAGGTTTATAGACATAGGAACCAGAG
CGCTTAAA
ATTTTCAGGGATGTTACCTCCATTAAAAAATTCAACAGCCTTTATATAAGGA
ACTTCTTC
TATAAAGAGAGAGGGGCTACCAGTTTCAAGAAGAGGGGATAATAGGGAAT
GGAGTTCAGA
GGGAGAACCGACAAACTCGCCTTGTGCTTCAATTTTATTTCGTTGCTTGGC
GAATAATTC
GATCGATGAAGTGAGACGTTCATCTATATAAGGTGCCCAGTTTTGCCACGCT
TGAAATGC
AGCAATAAAGTCTTCCCATTCCCATGTAATTGAGAAAATTGATACATTTTTTA
TCGGGTG
GACTCGAAAAGTTAAGGAAGTGACAATTCCGAAGTTTCCACCACCGCCAC
CACGGCATGC
CCAAAAAAGGTTAGAGTTTTCTTGTTCATTTGCACGAATGAGTTTTGCACC
AAATTTTCC
ACACGCTTGTACCATTTCCACTTCCATTAATTGATCACATGTTAATCCAAATA
AGCGCGA
AAGCATACCAATACCACCGCCAAGTGCTAATCCAACAACTCCAACGCTTGC
ACTTGTACC
AGCAGGAATTGTAACACCGTAATTCCAAAGTTCTTTATAAACAGTGCCAAG
ATTTGCACC
AGCCTCAATTGTTGCTGTTAATTTCTCTGTATTAACAGTAATGCGATGCATTT
CACTCAC
ATCAATAATAAGTCCTCTATTTAAAAGAGAAAAATTTTCATAGCTATGACGT

CCACTTCT
TAACCGAAATGGTATATGACGTTCACGTGCCCATTTTAAGGCGTTACAGACA
TCCTTGTT
ATTTTGACAAAAAACAATAATACAAGGGAGTTTTGGAATACTTAAATTTAAA
TTCATTCG
GGCTACGTCGTAGTCAGGATCTGTGGGAACAACGATACGACCTGTTAATTT
TGTTTGCTT
CAA
>00222|genome:54635-56170|strand:plus|identity:30.843%|evalue:3.57E-66|cover age:91%
GCCTGTTCTGTGACGACAAATACAACAAATGATCATAAAAATATGAATGATA
AAAAAACA
CTACAGACTGAAACAGCTACAACACCATTGAAAGTTGAAAAAGGACCAGA
AGTTACTTTA
ATAGCGAAAGAAGAAAAGCAAAAATTAAGTAACGGTGTTATTGTTCCAGTC
TGGACATTT
AATGGCTCATCTCCTGGTTCAGAAATTCGGGTGAAAAAAGGTGAAAAGGT
GAAAGTGACA
TTAAAAAATGAATTATCTGCACCAGTATCTATTCATTGGCATGGATATCCTGT
CCCAAAT
AACATGGATGGAATTCCAGGCGTGACACAAGATGCAGTTGAACCAGGAAA
AAGTTTCACT
TACGAATTTGAAGCGAACGTACCAGGAACGTACTGGTATCACTCGCATCAA
GATTCTGTA
AATCAATTAGATAGAGGTTTGTATGGAGCGCTCATTGTAGAAGATACAAAG
GAAAAATAT
GATAAAGATTACACATTAATGTTGGATGAATGGGTTACAGATAAAGAAGAA
ATGAATAAG
CAGTTAAAAGAAATGACAAAAGGGCAAATAGGTAATAAATCTAAAGGTAAT
GAAAATGGG
GAAAAGAATGATGATAAGAACGGCATGGCTCATTCTGATATGAACATGGGC
AGTGATAAA
AAAGACTCTAGTAATATGGAAGGAATGGACCATGGAAATATGAAGATGGAA
GGTCATGAT
ATGAGTATGTACGACTTATTCACAATCAATGGAAAAAGCGGTGATTTAGTAG
CGCCATTA
AAAGTGAATAAGGGAGATAACGTTCGTCTTAGACTCGTCAATGCTGGTTAT
CTATCACAT
GATATACATGTTCATGGCCATGATATAAAAGTAATTGCGACAGATGGTCAAC
CAATAAAC
GATCCAAAAGTTATAAAGGATAAAGTAATTTCAATCGCACCGGGTGAACGT
TATGATATT
GAATTTACTGCTAATAATCCTGGGAAATGGTATGTTGAAGACCATTCGAAAA
ATAAAGGT

GCAAAAGGAATGAAAGCTGTTATTGAGTATGATGGCAGCAAAGAGATGAA
AGATAAAGCA
GACGAAAAAGAAAAATTACCGAAAGTAGATATAATGAAATATGGTACTAAA
AAATTAGGT
AGTTTCACATTAAATCAAGAGTATACTGCCACATATAATATGGACTTAAATAC
GCAAATG
AATGGAAATGAAATGGTATATACAATTAACGGAAAGGTATTTCCGGATATTG
ACCCAATT
CAAGTAAAAAAGGGTGATTTAGTAAAAGTGAAGTTGGTAAATCGTTCTAAA
ATGGATGAT
CACCCAATGCATTTACACGGACACTTTTTCCAGGTGTTGAGTAAAGATGGA
AAACCGATA
GAAGGTTCTCCAATTGTGAAAGATACATTGAACTTAAAACCGGGAGAAGA
ATATGAAGTA
GCCTTTGTAGCAGATAATCCGGGCGATTGGATGTTCCACTGTCATGATTTAC
ACCATGCT
TCAGCTGGGATGGTAACAGAAGTAAAATATACAGAT
**>00223|genome:54743-55138|strand:plus|identity:34.586%|evalue:6.55E-20|cover
age:73%**
GAAGTTACTTTAATAGCGAAAGAAGAAAAGCAAAAATTAAGTAACGGTGTT
ATTGTTCCA
GTCTGGACATTTAATGGCTCATCTCCTGGTTCAGAAATTCGGGTGAAAAAA
GGTGAAAAG
GTGAAAGTGACATTAAAAAATGAATTATCTGCACCAGTATCTATTCATTGGC
ATGGATAT
CCTGTCCCAAATAACATGGATGGAATTCCAGGCGTGACACAAGATGCAGTT
GAACCAGGA
AAAAGTTTCACTTACGAATTTGAAGCGAACGTACCAGGAACGTACTGGTAT
CACTCGCAT
CAAGATTCTGTAAATCAATTAGATAGAGGTTTGTATGGAGCGCTCATTGTAG
AAGATACA
AAGGAAAAATATGATAAAGATTACACATTAATGTTG
